# Low Specific Phosphorus Uptake Affinity of Epilithon in Three Oligo- to Mesotrophic Post-mining Lakes

**DOI:** 10.3389/fmicb.2021.735498

**Published:** 2021-10-06

**Authors:** Eliška Konopáčová, Jiří Nedoma, Kateřina Čapková, Petr Čapek, Petr Znachor, Miloslav Pouzar, Milan Říha, Klára Řeháková

**Affiliations:** ^1^Institute of Environmental and Chemical Engineering, Faculty of Chemical Technology, University of Pardubice, Pardubice, Czechia; ^2^Institute of Hydrobiology, Biology Centre of the Czech Academy of Sciences, České Budějovice, Czechia; ^3^Faculty of Sciences, University of South Bohemia, České Budějovice, Czechia; ^4^Center of Materials and Nanotechnologies, University of Pardubice, Pardubice, Czechia

**Keywords:** epilithon, phosphorus uptake, specific P affinity, oligotrophic lakes, periphyton, hydric recultivation

## Abstract

Epilithon contributes to phosphorus (P) cycling in lakes, but its P uptake traits have been rarely studied. We measured the chemical composition of epilithon and its inorganic P uptake kinetics using isotope ^33^P in three deep oligo- to mesotrophic post-mining lakes in April, July, and October 2019. Over the sampling period, epilithon biomass doubled, while the P content in biomass dropped to 60% of the April values, and the seasonal changes in P content expressed per epilithon area were only marginal and statistically not significant. High epilithic C:P molar ratios (677 on average) suggested strong P deficiency in all investigated lakes. Regarding the kinetic parameters of phosphorus uptake, maximum uptake velocity (*V*_*max*_, seasonal range 1.9–129 mg P g OM^–1^ h^–1^) decreased by an order of magnitude from April to October, while half-saturation constant (*K*_*S*_, seasonal range 3.9–135 mg P L^–1^) did not show any consistent temporal trend. Values of epilithic specific P uptake affinity (*SPUA*_*E*_, seasonal range 0.08–3.1 L g OM^–1^ h^–1^) decreased from spring to autumn and were two to four orders of magnitude lower than the corresponding values for seston (*SPUA*_*sest*_), which showed an opposite trend. Considering our results, we suggest a possible mechanism underlying a stable coexistence of planktonic and epilithic microorganisms, with plankton prospering mostly in summer and autumn and epilithon in winter and spring season. Additionally, a phenomenon of reversible abiotic P adsorption on epilithon was observed.

## Introduction

Periphyton is an assemblage of autotrophic and heterotrophic organisms that produces extracellular polymeric substances, all together forming a biofilm attached to a solid substrate (Azim et al., 2005; Cantonati and Lowe, 2014). Epilithon is a subgroup of periphyton covering submerged surfaces of stones (Azim et al., 2005). Periphyton represents an important component of aquatic food webs and biodiversity (Vymazal and Richardson, 1995; Azim et al., 2005) and can contribute considerably to nutrient cycling (Dodds, 2003; Wyatt et al., 2019). Periphyton can account for up to 92% of the total algal primary production (production of phytoplankton and periphyton) in oligotrophic lakes (Vadeboncoeur and Steinman, 2002).

Periphyton productivity may be limited by the availability of inorganic nutrients, especially phosphorus (P), which is an essential constituent of microbial cells. Integrated into phospholipids, nucleic acids, coenzymes, and various metabolites, it is considered the most common element limiting the growth rates and metabolic activities of photoautotrophs in temperate freshwater ecosystems (Hudson et al., 2000; Grossman and Aksoy, 2018). Whereas P uptake by phytoplankton has been widely studied (Tanaka et al., 2006; Lindemann et al., 2016; Cáceres et al., 2019; Fitzsimons et al., 2020), only few studies have addressed the kinetics of P uptake by periphyton in lentic waters (Hwang et al., 1998; Scinto and Reddy, 2003), and no study ever quantified the P uptake of periphyton in temperate freshwaters. Given the above-mentioned importance of periphyton for total primary production of lakes, the periphyton P uptake might be significant as well. A study of P fluxes in the shallow subtropical Lake Okeechobee found a significantly greater P uptake in phytoplankton than in periphyton. However, in an oligotrophic marsh area in the lake, due to the well-developed periphytic biomass, the cumulative P uptake by periphyton was twice as high as P uptake by phytoplankton (Hwang et al., 1998).

A thorough analysis of periphyton and phytoplankton contributions to P cycling in lake ecosystems requires the determination of kinetic parameters of P uptake for both periphyton and phytoplankton. In osmotrophic organisms, phosphorus can only be taken up in a substantial amount in the form of inorganic orthophosphate (*P*_*i*_) (Cembella et al., 1984). The relationship between *P*_*i*_ concentration in water and its uptake rate can be often described by a hyperbolic function analogous to the Michaelis–Menten equation for enzymatic reaction kinetics possessing two parameters: maximum velocity (*V*_*max*_) and half-saturation constant (*K*_*S*_, concentration at which *V*_*max*_ = 1/2). However, at extremely low *P*_*i*_ concentrations, typical for oligotrophic waters, P uptake velocity is far below *V*_*max*_, as *P*_*i*_ is much smaller than *K*_*S*_ (Taylor and Lean, 1991; Chen and Taylor, 2011). A relevant parameter describing P uptake and the competitive ability of microorganisms at low *P*_*i*_ is the specific P uptake affinity (*SPUA*), calculated as a proportion of *V*_*max*_ and *K*_*S*_, equal to the slope of the velocity vs. concentration at the initial linear region of the curve (Healey, 1980; Button, 1985). Interpretation of the *SPUA* values is not straightforward due to their unusual units (L mg OM^–1^ h^–1^); however, it is analogous to the clearance rate of zooplankton—the volume of water cleared of phosphate per unit biomass per unit time (Thingstad and Rassoulzadegan, 1999).

Large lakes newly emerge during the hydric recultivation of post-mining pits. Understanding the nutrient cycling and energy fluxes in the unique ecosystems of post-mining lakes is urgently needed since their number is expected to rise in the upcoming decades due to the coal mining suppression associated with the European Union’s Green Deal (Larondelle and Haase, 2012; European Commission, 2018). Post-mining lakes are often oligotrophic with very low P concentrations (total phosphorus, *TP* < 15 μg L^–1^ and soluble reactive phosphorus concentration, *SRP* ∼ 1 μg L^–1^) (Schultze et al., 2010). However, no detailed report on P cycling in these anthropogenic lakes and about the periphytic organisms involved is available.

In the present study, we sampled three recently flooded oligo- to mesotrophic post-mining lakes in the north-west region of the Czech Republic (Milada, Medard, and Most Lakes) to evaluate the role of dissolved inorganic phosphorus acquisition for periphyton nutrition and in-lake nutrient cycling. Despite very low P concentration in water, dense mats of periphyton, mainly represented by epilithon, have developed in all investigated lakes (Bešta et al., in prep).

We sought to investigate P uptake traits of epilithon in these three lakes during the vegetation season, using ^33^P-labelled orthophosphate. Our specific objectives were: (i) to determine kinetic parameters of the epilithon P uptake, namely, the specific P uptake affinity (*SPUA*_*E*_), maximum P uptake velocity (*V*_*max*_), and half-saturation constant (*K*_*S*_); (ii) to decipher the environmental factors affecting the specific P uptake affinity; and (iii) to compare specific P uptake affinities and doubling times of epilithon and seston.

## Materials and Methods

### Study Sites

**FIGURE 1:**
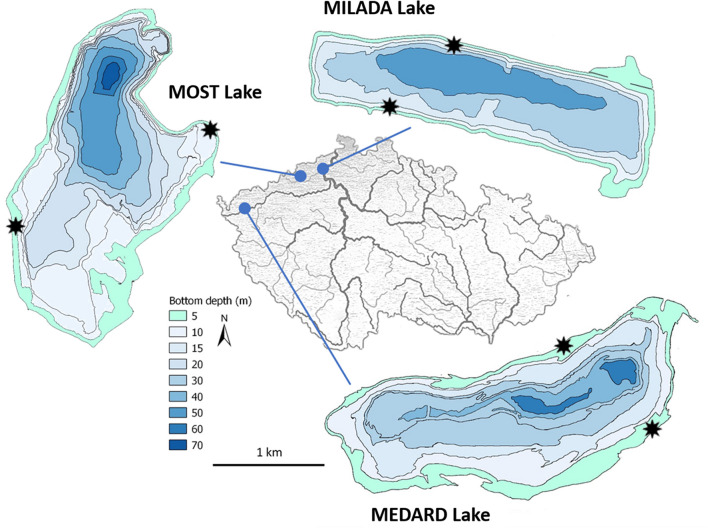
Maps of the investigated post-mining lakes and location of the sampling sites (asterisks).

Three oligo- to mesotrophic post-mining lakes, Milada (area: 252 ha, max/average depth: 25/16 m, flooded in 2010), Medard (493 ha, 59/28 m, flooded in 2016), and Most (311 ha, 75/22 m, flooded in 2014), situated in the north-western Bohemia, the Czech Republic, were chosen as study sites (Figure 1 and Supplementary Figures 7–9). All lakes possess well-developed epilithon assemblages in their littoral zone (Supplementary Figure 10). Basic limnological parameters of the lakes are summarised in Table 1.

**TABLE 1:** Chemical and limnological parameters of the three post-mining lakes in the Czech Republic sampled from the depth of 0.5 m at their deepest points in 2019.

**Lake**	**Month**	**T_*w*_ (°C)**	**pH**	**Conductivity (μS cm^–1^)**	***TP* (μg L^–1^)**	***SRP* (μg L^–1^)**	**Si (mg L^–1^)**	**NO_3_ (mg L^–1^)**	**DOC (mg L^–1^)**	***Chla* (μg L^–1^)**	**Z_*mix*_ (m)**	**Z_*eu*_ (m)**
Milada	April	7.7	9.1	1,037	17.1	3.2	0.8	0.13	6.8	8.2	∼8*	9.9
	July	22.4	9.1	1,030	10.2	1.6	0.9	0.02	8.1	2.1	6	11.7
	October	14.8	9.1	1,035	13.9	1.5	0.7	0.02	8.9	4.3	10	15.0
Medard	April	7.5	7.7	1,093	4.5	1.0	2.7	1.26	3.0	0.6	∼4*	9.4
	July	21.9	7.9	1,080	4.0	b.d.l.	2.9	1.12	3.1	1.0	7	17.6
	October	12.7	7.8	1,087	4.0	b.d.l.	2.4	1.12	3.1	1.2	12	14.5
Most	April	11.7	8.6	540	7.9	1.6	1.2	0.92	4.6	1.0	5	16.7
	July	24.1	8.7	524	13.7	2.0	0.7	0.64	4.6	1.5	3	13.6
	October	17.0	8.8	533	14.2	2.1	0.5	0.56	5.0	3.1	10	14.6

Tw, surface water temperature; *SRP*, soluble reactive phosphorus; Si, dissolved reactive silica; NO_3_ nitrate-nitrogen; DOC, dissolved organic carbon; *Chla*, chlorophyll *a*; Z_*mix*_, mixed layer; Z_*eu*_, euphotic depth—the depth receiving 1% of the surface photosynthetically active radiation; b.d.l., below the detection limit of 0.5 μg L^–1^ for SRP. *Unstable stratification.

### Sampling

In each lake, epilithon was sampled at two opposite shores of each lake, designated as North (N) and South (S) (see Figure 1 and Supplementary Table 4 for more details). Twelve small epilithon-covered stones (approx. diameter 4 cm) were randomly collected by scuba diving at each site from 0.5 and 1.5 m depths in April, July, and October 2019. Each stone was placed into a plastic container (volume 150 mL) filled with lake water. Epilithon samples were kept in the lake water at *in situ* temperature and a photoperiod of 16:8 (light:dark) until the P uptake experiments (<2 days). Lake water for chemical analyses and epilithon P uptake experiments was collected with a Friedinger sampler above the deepest points of the lakes from the depth of 0.5 m and stored in the dark at 4°C until analysis. The cell-free filtrate of lake water was prepared by passing it through a polycarbonate filter (0.22-μm porosity; Sterlitech Corporation, Seattle, WA, United States) and stored at 4°C until the start of the experiment (<2 days).

### Background Limnological Parameters

Vertical profiles of water temperature, pH, and O_2_ concentration were measured with YSI EXO 2 multiparametric probe (YSI Inc., Yellow Springs, OH, United States). The euphotic depth was estimated as the depth of 1% of surface irradiance calculated from vertical light profiles obtained using LICOR LI-1400 datalogger with a spherical quantum underwater sensor LI 193 SA (LI-COR, Lincoln, NE, United States). Chlorophyll *a* (*Chla*) was determined according to ISO 10260:1992 (ISO, 1992); total organic carbon (*TOC*) and total nitrogen (*TN*) were determined using Shimadzu *TOC*/*TN* analyser (Shimadzu Corp., Kyoto, Japan). Total phosphorus (*TP*) was measured spectrophotometrically after nitric-perchloric acid digestion (Kopáček and Hejzlar, 1993) using Flow Injection Analyser. Dissolved reactive silica (*DSi*) (Mackereth et al., 1978) and soluble reactive phosphorus (*SRP*) (Murphy and Riley, 1962) were determined spectrophotometrically. Nitrate-nitrogen (*NO_3_–N*) was determined following the procedure by Procházková (1959).

### Epilithon Chemical Characterisation

After the termination of ^33^P uptake experiments (see below), epilithon was quantitatively removed from each stone by scalpel and toothbrush and dried at 110°C to constant weight, and the dry weight (DW) was determined. To determine the organic matter (OM) content, the dry sample was combusted in a muffle furnace at 450°C for 12 h. For the determination of epilithon elemental (C, N, and P) composition and biomass (DW and OM per area), we used different stones than those used for uptake experiments, albeit collected from the same sampling site. Samples were frozen at –20°C till the analysis and homogenised using glass beads and an automatic homogeniser. *TN, TOC*, and *TP* were analysed, as described above.

### Epilithon P Uptake Kinetics

Before each experiment, the lake water was removed from containers containing epilithon-covered stones. Stones were gently rinsed with 50 mL of the 0.2-μm filtered (cell-free) lake water, and 100 mL of the filtered water was added. The containers were supplemented with five different concentrations of orthophosphate (*P*_*add*_, 1, 3, 10, 30, and 100 mg P L^–1^, added as KH_2_PO_4_) in duplicates. Incubations started by the addition of 20 μL of carrier-free ^33^P-labelled H_3_PO_4_ in the tracer amount (50–100 kBq; American Radiolabeled Chemicals, Inc., St. Louis, MO, United States) along with unlabelled orthophosphate into testing containers placed in a gently shaken (120 rpm) water bath tempered at *in situ* temperature of investigated lakes. Samples were incubated for 180 min under laboratory illumination (cca 10 μE m^–2^ s^–1^ PAR). At 15–30 min intervals, 0.6-mL aliquots were transferred into Eppendorf tubes containing 0.6 mL of scintillation cocktail (Packard Ultima Gold) and vortexed vigorously. Radioactivity (typically 20,000–40,000 c.p.m. per aliquot) was determined with a liquid scintillation counter (Tri-Carb 2900TR, Packard, Conroe, TX, United States).

P incorporation into the epilithon biomass was considered to be proportional to the ^33^P disappearance from incubation water, as confirmed in an experiment with simultaneous spectrophotometric and scintillation detection of dissolved P (not shown). The P uptake rate constant, *k*_*upt*_ (h^–1^), which reflects the initial uptake rate, was calculated applying the second-order polynomial function (Currie and Kalff, 1984):


(1) $$A_{t}=A_{0}+A_{1}\times t+A_{2}\times t^{2},$$

where *t* (hours) is time; *A*_*t*_ is ^33^P activity (c.p.m.) at the time *t*; and *A*_0_, *A*_1_, and *A*_2_ are parameters of the polynomial function fitted with non-linear regression; the ratio of *A*_1_ to *A*_0_ equals *k*_*upt*_. The second-degree polynomial function was used here to estimate the P uptake rate at time zero. The polynomial function can be used as an approximation of any differentiable function, and its second term then defines the slope of decrease of that function at time zero. Only the points where *A*_*t*_/*A*_0_ > 0.3 were included in the calculations (Currie and Kalff, 1984).

The velocity of P uptake by epilithon, *v*_*upt*_ (mg P g OM^–1^ h^–1^), was calculated as follows:


(2) $$v_{u\,p\,t}=\frac{k_{u\,p\,t}\times P_{a\,d\,d}\times V_{i\,n\,c}}{O\,M},$$

where *V*_*inc*_ (L) is incubation volume and OM (g) is organic matter content in the epilithon sample. To study concentration dependence of the P uptake velocity, it was plotted as a function of *P*_*add*_ and fitted either to a Michaelis–Menten model:


(3) $$v_{u\,p\,t}=\frac{V_{m\,a\,x}\times P_{a\,d\,d}}{K_{S}+P_{a\,d\,d}},$$

or, if the P uptake velocity did not show saturation, to a simplified linear model:


(4) $$v_{u\,p\,t}=v_{s\,l\,o\,p\,e}\times P_{a\,d\,d},$$

where *V*_*max*_ (the maximum P uptake velocity; mg P g OM^–1^ h^–1^) and *K*_*S*_ (half-saturation constant; mg P L^–1^) are the parameters of the Michaelis–Menten model (Eq. 3); the linear model (Eq. 4) had only one parameter, *v*_*slope*_ (L g OM^–1^ h^–1^). The more complex model (Eq. 3) was selected in preference to the simpler one (Eq. 4) when improving the fit at the probability level of *p* < 0.05 (*F* test). Parameter estimations and model selection were performed in PRISM v.7 (GraphPad Software). For saturation data (Eqs 3, 4), relative weighting by 1/(*v*_*upt*_)^2^ was applied, as the error of the *v*_*upt*_ was heteroscedastic, accounting for approximately 30% (average difference between duplicates), irrespectively of *P*_*add*_. No weighting was applied to the time-course data. Outliers were excluded with the PRISM v.7 built-in algorithm using the recommended settings (Motulsky and Brown, 2006). We further used—assuming the Michaelis–Menten uptake kinetics—an alternative approach of kinetic parameters (^^*^V_*max*_ and ^^*^K_*S*_) estimation based on fitting the integrated form of the Michaelis–Menten equation directly to ^33^P disappearance data across all concentrations of *P*_*add*_ (see Supplementary Material).

Epilithon specific P uptake affinity, *SPUA*_*E*_ (L g OM^–1^ h^–1^), for the Michaelis–Menten model (Eq. 3) was calculated as:


(5) $$S\,P\,U\,A_{E}=\frac{V_{m\,a\,x}}{K_{S}},$$

while for the linear model (Eq. 4), it was directly equal to *v*_*slope*_.

Epilithon P doubling time, *PDT*_*E*_ (h), i.e., the theoretical time necessary for doubling of the actual epilithon P content, was calculated as:


(6) $$P\,D\,T_{E}=\frac{E\,P\,C}{\left(S\,P\,U\,A_{E}\times P_{i}\right)},$$

where *EPC* (μg P g OM^–1^) is epilithon phosphorus content and *P*_*i*_ is concentration of (bioavailable) dissolved inorganic phosphorus. As *P*_*i*_ cannot be reliably determined in lake water under conditions of P deficiency (see section “Discussion”), two scenarios were applied: (1) *P*_*i*_ = *SRP* (9.6–102 nmol L^–1^, assuming 0.3 μg L^–1^ for *SRP* values below the detection limit) and (2) *P*_*i*_ = 0.1 μg L^–1^ (3.2 nmol L^–1^).

### Abiotic P Adsorption

To distinguish the P uptake by cells from the abiotic P adsorption, two samples with formaldehyde (final conc. 4%) were pre-incubated overnight (>12 h) in each experiment. These samples received the lowest concentration of *P*_*add*_ used (i.e., 1 mg L^–1^), and the effect of formaldehyde was expressed relative to uninhibited uptake velocity. Additional experiments were performed to elucidate concentration dependency of the abiotic adsorption over a wide range of P concentrations (*P*_*add*_ = 0.01, 0.1, 1, and 10 mg P L^–1^), and reversibility of the abiotic P adsorption using oxalacetate-based surface wash reagent (for composition, see Supplementary Table 3) (Tovar-Sanchez et al., 2003; Yao et al., 2011) applied immediately after termination of ^33^P uptake experiments for 1 h. The amount of ^33^P released by the surface wash reagent was expressed relative to the ^33^P removed from the water in the presence of 4% formaldehyde.

### Seston P Uptake Kinetics

Lake-water samples (30 mL) were supplemented simultaneously with 50–100 kBq of carrier-free ^33^P-labelled H_3_PO_4_ in the tracer amount and seven concentrations of unlabelled orthophosphate (*P*_*add*_, range 0.1–60 μg P L^–1^, added as KH_2_PO_4_) and incubated at *in situ* temperature for 180 min. At appropriate time intervals (2–30 min), 0.5-mL subsamples were filtered through polycarbonate filters (Poretics, 0.2 μm of mean porosity). The filter-retained activity was measured with the scintillation counter. The P uptake rate constants, *k*_*upt*_, were determined from the time courses of ^33^P incorporation with the method of Currie and Kalff (1984), applying the second-order polynomic equation:


(7) $$\frac{P_{t}}{T}=P_{0}+k_{u\,p\,t}\times t+a\times t^{2},$$

where *P*_*t*_ is filter-retained ^33^P activity (c.p.m.) at time *t*, *T* is total ^33^P activity added, and *P*_0_ is filter-retained ^33^P activity at zero time, to the initial part of data (*P*_*t*_/*T* < 0.7) (Currie, 1990). The P uptake velocity, *v*_*upt*_ (μg P L^–1^ h^–1^), was calculated as:


(8) $$v_{u\,p\,t}=k_{u\,p\,t}\times P_{a\,d\,d}.$$

The maximum velocity of P uptake, *V_*max*_sest* (μg P L^–1^ h^–1^), and half-saturation constant, *K_*S*_sest* (μg P L^–1^), were determined by non-linear regression as described above (Eq. 3).

To compare specific P uptake affinities of epilithon and seston, expressed in the same units, the seston specific P uptake affinity, *SPUA*_*sest*_ (L g OM^–1^ h^–1^), was calculated as:


(9) $$S\,P\,U\,A_{s\,e\,s\,t}=\frac{V_{m\,a\,x}\,s\,e\,s\,t}{K_{S}\,s\,e\,s\,t\times O\,M\,s\,e\,s\,t},$$

where OM is seston organic matter content (g L^–1^) estimated from *Chla* concentration in lake water assuming OM*:Chla* (w/w) ratio of 200 (Yacobi and Zohary, 2010).

In order to compare epilithon and seston P doubling times, seston P doubling time, *PDT*_*sest*_ (h), was calculated—assuming exponential growth—for both above defined *Pi* scenarios as:


(10) $$P\,D\,T_{s\,e\,s\,t}=\frac{l\,n\,\left(2\right)\times S\,P\,C}{\left(S\,P\,U\,A_{s\,e\,s\,t}\times P_{i}\right)},$$

where *SPC* is seston P content estimated as *TP - SRP*. Note that assuming the same *P*_*i*_ for seston and epilithon (homogeneous *P*_*i*_ distribution in epilimnion), the ratio *PDT*_*sest*_/*PDT*_*E*_ does not depend on *P*_*i*_.

Turnover time of dissolved orthophosphate, *TT-PO_4_* (h), was determined as 1/*k*_*upt*_ measured in the absence of added P and used for epilimnion P deficiency assessment (Istánovics et al., 1992).

## Results

### Background Limnological Parameters

All lakes had high water transparency corresponding to the euphotic depth (the depth of 1% of the surface irradiance) between 10 and 18 m (Table 1) and were stratified with the mixed depth varying from 3 to 11 m. In April, the sampling was performed shortly after the spring overturn; thus, the stratification was weak, nutrient concentrations were higher, and the temperature was lower (7–11°C) than in summer and autumn (13–24°C, Table 1). Lakes Medard and Most were characterised by low *Chla* and *TP* concentrations (0.9 and 1.9 μg L^–1^; and 4.2 and 11 μg L^–1^, respectively, in their seasonal averages). Lake Milada’s *Chla* and *TP* concentrations were slightly higher (5.1 and 14.1 μg L^–1^, respectively).

### Epilithon Characterisation

**FIGURE 2:**
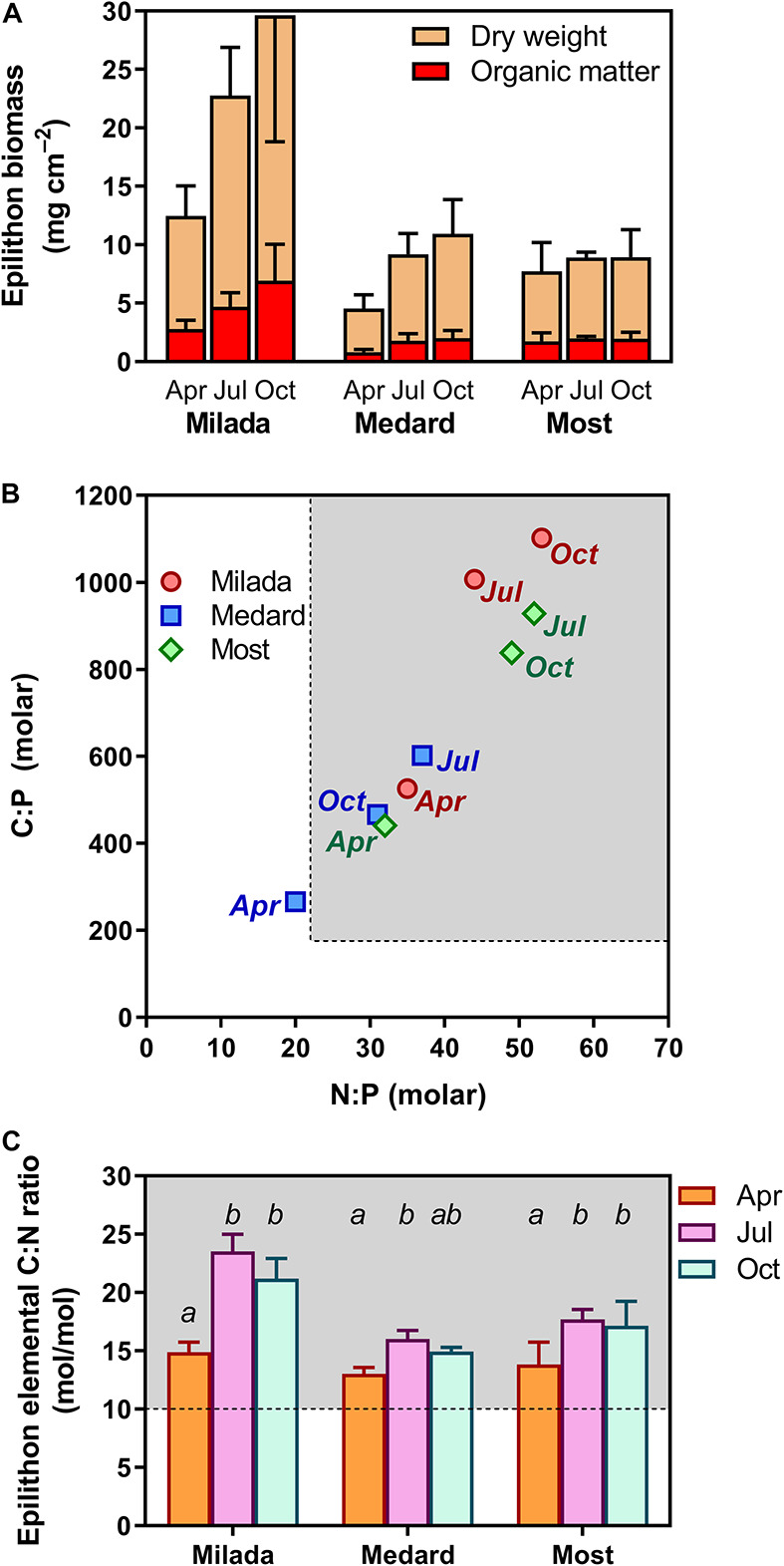
Biomass and elemental composition of epilithon in three post-mining lakes sampled in the Czech Republic in 2019. **(A)** Epilithon dry weight and organic matter content expressed per overgrown area, **(B)** relationship between epilithon molar N:P and C:P molar ratios (error bars omitted for the sake of clarity), and **(C)** epilithon C:N ratios. Columns show averages of 4 (2 × 2) values from two sampling sites and two depths ± SD. Different lowercase letters indicate significant seasonal differences within lakes (*p* < 0.05, two-way ANOVA with Tukey’s multiple comparisons post-test). Shaded areas **(B,C)** show values indicating P and N limitation, respectively, given by Hillebrand and Sommer (1999).

Parameters related to epilithon biomass and chemical composition, as well as their differences among lakes and seasonal development with statistical differences, are summarised in Figure 2 and Table 2 (for the full two-way ANOVA table, see Supplementary Table 1). Epilithon biomass expressed as OM content and normalised per area on average doubled from April (1.8 mg cm^–2^) to October (3.6 mg cm^–2^), being about three times higher in Lake Milada (4.8 mg cm^–2^) than in the two other lakes (Table 2 and Figure 2A); the corresponding dry-weight figures showed a similar pattern (Table 2 and Figure 2A). Average biomass P content dropped to ∼60% from April (3.6 mg P g OM^–1^) to October (2.2 mg P g OM^–1^) independently of the lake (Table 2). In contrast, average epilithic P content per area considerably differed among the lakes, being 2.5 times higher in Milada Lake (11 μg P cm^–2^) than in the two other lakes but showing only marginal and statistically insignificant seasonal trends that were inconsistent among lakes.

**TABLE 2:** Average values of epilithon biomass- and P uptake-related variables measured in three post-mining lakes in the Czech Republic in 2019.

	**Lake averages**				**Sampling-season averages**			
**Epilithon variable**	**Milada**	**Medard**	**Most**	***p***	**April**	**July**	**October**	***p***
*V*_*max*_ (mg P g OM^–1^ h^–1^)	22.3^*a*^	11.5^*a*^	30.0^*a*^	0.4392	59.0^*a*^	7.0^*b*^	6.6^*b*^	**<0.0001**
*K*_*S*_ (mg P L^–1^)	21.3^*a*^	19.8^*a*^	34.1^*a*^	0.9593	54.0^*a*^	11.3^*a*^	20.1^*a*^	0.0552
Specific P uptake affinity (L g OM^–1^ h^–1^)	0.65^*a*^	0.60^*ab*^	0.83^*b*^	0.0051	1.14^*a*^	0.69^*b*^	0.25^*c*^	**<0.0001**
Biomass (mg OM cm^–2^)	4.80^*a*^	1.54^*b*^	1.89^*b*^	**<0.0001**	1.79^*a*^	2.65^*ab*^	3.63^*b*^	**0.0042**
Biomass (mg DW cm^–2^)	16.7^*a*^	6.7^*b*^	6.6^*b*^	**<0.0001**	6.4^*a*^	10.1^*ab*^	12.9^*b*^	**0.0036**
OM/DW	0.29^*a*^	0.23^*b*^	0.29^*a*^	**0.0001**	0.27^*a*^	0.26^*a*^	0.28^*a*^	0.5668
P content (mg P g OM^–1^)	2.49^*a*^	3.14^*a*^	2.42^*a*^	0.058	3.64^*a*^	2.21^*b*^	2.18^*b*^	**0.0001**
P content (μg P cm^–2^)	10.9^*a*^	4.3^*b*^	4.4^*b*^	**<0.0001**	6.0^*a*^	5.8^*a*^	7.4^*a*^	0.5314
C:P (molar ratio)	866^*a*^	444^*b*^	736^*a*^	**0.0001**	411^*a*^	831^*b*^	802^*b*^	**<0.0001**
C:N (molar ratio)	19.5^*a*^	14.7^*b*^	16.2^*c*^	**<0.0001**	13.9^*a*^	18.7^*b*^	17.8^*b*^	**<0.0001**
N:P (molar ratio)	43.8^*a*^	29.4^*b*^	44.4^*a*^	**0.0086**	29.2^*a*^	44.4^*b*^	44.1^*b*^	**0.0075**

*Differences were tested with two-way ANOVA, with Lake and Sampling season as factors. Different lowercase letters indicate significant difference between lakes or sampling seasons (*p* < 0.05; Tukey’s multiple comparisons post-test). For full ANOVA table, see Supplementary Table 1. Data with variability exceeding one order of magnitude were log-transformed.*

*V_*max*_, maximum P uptake velocity; *K*_*S*_, half-saturation constant; OM, organic matter; DW, dry weight; p, significance of the main effect.*

*Values in bold indicate statistically significant results.*

Epilithon nutrient molar element ratios were generally high (Table 2)—C:P molar ratios ranged from 160 to 1,260, N:P molar ratios ranged from 13 to 68 (Figure 2B), and C:N molar ratios ranged from 12 to 25 (Figure 2C), being on average 1.3–2.0 times lower in Lake Medard than in the two other lakes (Table 2 and Figures 2B,C). All the ratios were significantly (up to two-fold) higher in June and October than in April (Table 2 and Figures 2B,C).

### Epilithon P Uptake

**FIGURE 3:**
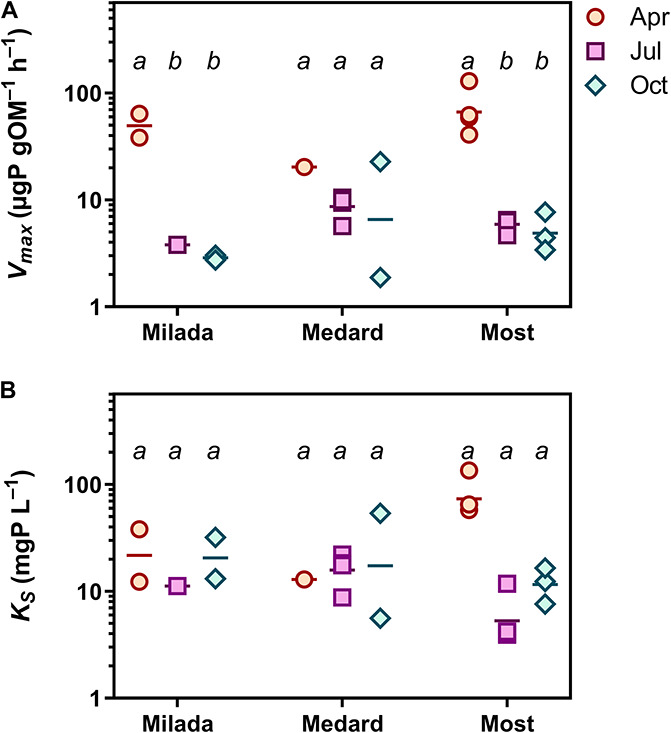
Epilithon P uptake in three post-mining lakes sampled in the Czech Republic in 2019. **(A)** Maximum P uptake capacity, *V*_*max*_. **(B)** Half-saturation constant, *K*_*S*_. Symbols in two lower panels show individual values. Different lowercase letters indicate significant seasonal differences within lakes (*p* < 0.05, two-way ANOVA and Tukey’s multiple comparisons post-test).

**FIGURE 4:**
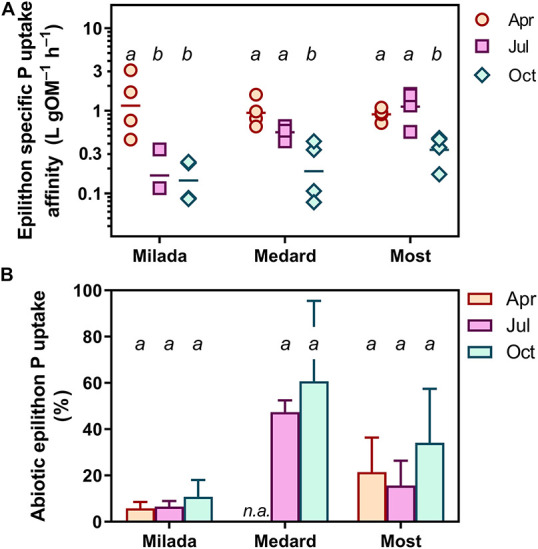
P uptake parameters of epilithon in three post-mining lakes sampled in the Czech Republic in 2019. Symbols show individual values; different lowercase letters indicate significant seasonal differences within lakes (*p* < 0.05; two-way ANOVA and Tukey’s multiple comparisons post-test). **(A)** Epilithon specific phosphorus uptake affinity (*SPUA*_*E*_). **(B)** Abiotic P uptake (adsorption). Columns show averages of four values from two sampling sites and two depths ± SD.

There was no systematic variability in any epilithon P uptake parameter within lakes (north shore vs. south shore; a depth of 0.5 vs 1.5 m; paired *t*-tests, *p* = 0.12–0.81). Hence, all these in-lake values were treated as replicates (*n* = 4; see, e.g., Figures 3, 4). Time course data on P uptake by epilithon (i.e., ^33^P disappearance from water) fitted well to second-order polynomial model (Supplementary Figure 1 and Table 2). Fourteen out of the 348 progress curves were excluded due to a lack of fit (high scatter or an increasing trend in the data). Calculated P uptake velocities (*v*_*upt*_) varied widely from 0.015 to 75 mg P g OM^–1^ h^–1^ and were strongly concentration-dependent (Supplementary Figures 2–4). The relationship between the concentration of added orthophosphate, *P*_*add*_, and *v*_*upt*_ followed the Michaelis–Menten kinetics in 23 out of the 35 cases, while the remaining 12 followed the simplified linear model (Table 3 and Supplementary Figures 2–4). Maximum P uptake velocity (*V*_*max*_) ranged between 1.9 and 129 mg P g OM^–1^ h^–1^ and significantly decreased over the season by around one order of magnitude. The corresponding estimates of the half-saturation constant (*K*_*S*_) ranged from 3.9 to 135 mg P L^–1^ with no seasonal trend (Table 3 and Figure 3). Both *V*_*max*_ and *K*_*S*_ did not significantly differ between lakes.

**TABLE 3:** A summary of estimates of kinetic parameters of P uptake by epilithon in three post-mining lakes in the Czech Republic in 2019 with indices of the goodness of fit.

**Date**	**Lake**	**Site**	**Depth (m)**	**Best model**	***F*-test *p***	***R* ^2^_*adj*_**	***V*_*max*_ (mg P g OM^–1^ h^–1^)**	***K*_*S*_ (mg P L^–1^)**	***SPUA*_*E*_ (L g OM^–1^ h^–1^)**
Apr 15, 2019	Milada	N	0.5	L	0.1304	0.6897	–	–	0.76 ± 0.099
Apr 15, 2019	Milada	N	1.5	MM	0.0043	0.5802	38.2 ± 16.1	12.3 ± 8.4	3.101 ± 1.178
Apr 15, 2019	Milada	S	0.5	L	0.0671	0.2854	–	–	0.448 ± 0.075
Apr 15, 2019	Milada	S	1.5	MM	0.0116	0.7822	63.8 ± 23.1	38.1 ± 18.4	1.674 ± 0.316
Jul 22, 2019	Milada	N	0.5	L	>0.9999	0.1619	–	–	0.116 ± 0.023
Jul 22, 2019	Milada	N	1.5	L	0.093	–0.001	–	–	0.113 ± 0.024
Jul 22, 2019	Milada	S	0.5	MM	0.0016	0.7033	3.8 ± 1	11.2 ± 4.8	0.34 ± 0.083
Oct 14, 2019	Milada	N	0.5	L	>0.9999	0.3484	–	–	0.088 ± 0.023
Oct 14, 2019	Milada	N	1.5	MM	0.0016	0.8029	3 ± 0.7	13.1 ± 5	0.232 ± 0.048
Oct 14, 2019	Milada	S	0.5	MM	0.0009	0.9658	2.7 ± 0.5	32 ± 7.2	0.085 ± 0.007
Oct 14, 2019	Milada	S	1.5	L	0.0012	–1.713	–	–	0.239 ± 0.099
Apr 8, 2019	Medard	N	0.5	L	–	0.5762	–	–	0.806 ± 0.153
Apr 8, 2019	Medard	N	1.5	L	0.1053	0.7249	–	–	0.643 ± 0.073
Apr 8, 2019	Medard	S	0.5	MM	0.0012	0.7103	20.3 ± 6.1	12.9 ± 6.2	1.567 ± 0.412
Apr 8, 2019	Medard	S	1.5	L	>0.9999	–0.861	–	–	0.979 ± 0.21
Jul 8, 2019	Medard	N	0.5	MM	0.0014	0.554	5.7 ± 1.8	8.7 ± 4.9	0.651 ± 0.219
Jul 8, 2019	Medard	N	1.5	MM	0.0002	0.979	9.4 ± 1.1	22 ± 3.9	0.427 ± 0.035
Jul 8, 2019	Medard	S	0.5	MM	0.0012	0.8483	10.5 ± 2.5	18.3 ± 6.2	0.574 ± 0.086
Jul 8, 2019	Medard	S	1.5	MM	0.001	0.8102	10 ± 2.4	17.5 ± 6.6	0.569 ± 0.115
Oct 21, 2019	Medard	N	0.5	L	0.1029	0.1392	–	–	0.106 ± 0.027
Oct 21, 2019	Medard	N	1.5	MM	0.0429	0.5773	1.9 ± 0.8	5.6 ± 4	0.336 ± 0.139
Oct 21, 2019	Medard	S	0.5	MM	0.0194	0.8056	22.8 ± 8.9	53.6 ± 26.7	0.425 ± 0.072
Oct 21, 2019	Medard	S	1.5	L	0.0589	0.4593	–	–	0.078 ± 0.02
Apr 29, 2019	Most	N	0.5	MM	0.0281	0.8046	40.8 ± 18.8	57.7 ± 34.4	0.708 ± 0.147
Apr 29, 2019	Most	N	1.5	MM	0.0059	0.8325	58.7 ± 20.3	64.9 ± 27.8	0.905 ± 0.121
Apr 29, 2019	Most	S	0.5	MM	0.0213	0.9262	129.2 ± 52.5	135.3 ± 62.9	0.955 ± 0.093
Apr 29, 2019	Most	S	1.5	MM	0.012	0.8296	62.1 ± 24.1	56.9 ± 27.8	1.092 ± 0.177
Jul 29, 2019	Most	N	0.5	MM	0.0086	0.659	6.5 ± 2.2	11.7 ± 6.4	0.554 ± 0.171
Jul 29, 2019	Most	N	1.5	MM	<0.0001	0.6611	6.3 ± 1.1	3.9 ± 1.5	1.594 ± 0.431
Jul 29, 2019	Most	S	0.5	MM	0.0008	0.7495	4.7 ± 1	4 ± 1.8	1.155 ± 0.341
Jul 29, 2019	Most	S	1.5	MM	<0.0001	0.7113	6.5 ± 1.2	4.2 ± 1.7	1.54 ± 0.435
Sep 30, 2019	Most	N	0.5	MM	0.0019	0.7695	4.4 ± 1.2	12.4 ± 5.3	0.359 ± 0.085
Sep 30, 2019	Most	N	1.5	MM	0.0016	0.7875	7.7 ± 2	16.5 ± 6.5	0.468 ± 0.095
Sep 30, 2019	Most	S	0.5	MM	0.0058	0.6378	3.4 ± 1	7.6 ± 3.9	0.448 ± 0.133
Sep 30, 2019	Most	S	1.5	L	0.0594	–0.1873	–	–	0.169 ± 0.038

*Numbers represent best-fit parameter ± standard error.*

*V_*max*_, maximum P uptake velocity; *K*_*S*_, half-saturation constant; SPUA_*E*_, specific P uptake affinity; OM, organic matter; N and S, the north and south sampling site, respectively; Best model, the most parsimonious model selected with F-test; MM, Michaelis–Menten kinetics (Eq. 3); L, linear model (Eq. 4).*

Fitting integrated form of the Michaelis–Menten equation to data across all concentrations of added P resulted in roughly similar estimates of kinetic parameters (^^*^V_*max*_, ^^*^K_*S*_, and their ratio, ^^*^SPUA_*E*_) as compared with estimates provided by Eqs 2, 3 (Supplementary Figure 14 and Table 5). However, the integrated Michaelis–Menten equation showed substantial lack of fit to the measured data in several instances especially at higher *P*_*add*_ concentrations since the P uptake rate did not exhibit any saturation trend (Supplementary Figures 11–13). For that reason, there was a poor correlation between the values of some corresponding kinetic parameters estimated by two independent approaches (r^2^ values resulting from log–log linear regression were 0.85, 0.54, and 0.13 for *V*_*max*_, *K*_*S*_, and *SPUA*_*E*_, respectively). Therefore, the approach based on determination of initial uptake rate (Eq. 1) followed by fit to either the Michaelis–Menten or simplified linear model (Eqs 3, 4) was chosen for further calculations.

Specific P uptake affinity (*SPUA*_*E*_) ranged from 0.08 to 3.1 L g OM^–1^ h^–1^ (Table 3 and Figure 4A). There was a significant seasonal decrease in average *SPUA*_*E*_ from 1.14 to 0.25 L g OM^–1^ h^–1^ independently of the lake (Table 2). The generalised linear mixed-effect model with season and lake treated as random factors indicated the C:N molar ratio of the epilithon biomass (*p* < 0.001) and the lake-water pH (*p* < 0.002) as significant drivers of *SPUA*_*E*_.

Epilithon P doubling time (*PDT*_*E*_), i.e., the theoretical time necessary for doubling the actual P content in the biomass assuming the actual *in situ* P uptake velocity, was rather long, ranging from 9.3 to 5,140 days or from 372 to 19,600 days, assuming *P*_*i*_ concentration equal to measured *SRP* or its assumed concentration 0.1 μg L^–1^, respectively (Table 4).

**TABLE 4:** A summary of estimates of phosphorus doubling times in epilithon (*PDT*_*E*_) and seston (*PDT*_*sest*_) in three post-mining lakes in the Czech Republic in 2019.

**Date**	**Lake**	***PDT*_*E*_ (days) *P*_*i*_ = *SRP***	***PDT*_*E*_ (days) *P*_*i*_ = 0.1 μg L^–1^**	***PDT*_*sest*_ (h) *P*_*i*_ = *SRP***	***PDT*_*sest*_ (h) *P*_*i*_ = 0.1 μg L^–1^**	**PDT ratio (*PDT*_*E*_ (h)/*PDT*_*sest*_ (h))**
Apr 15, 209	Milada	47 ± 36	1,500 ± 1,146	7.7	247.2	146 ± 111
Jul 22, 2019	Milada	396 ± 154	6,330 ± 2,470	0.5	7.8	19,358 ± 7,553
Oct 14, 2019	Milada	362 ± 149	5,435 ± 2,235	0.8	11.6	11,274 ± 4,636
Apr 8, 2019	Medard	183 ± 54	1,827 ± 539	51.3	512.7	86 ± 25
Jul 8, 2019	Medard	626 ± 246	1,878 ± 737	4.1	12.2	3,690 ± 1,449
Oct 21, 2019	Medard	2,841 ± 2,597	8,522 ± 7,791	2.5	7.5	27,265 ± 24,927
Apr 29, 2019	Most	105 ± 28	1,673 ± 455	4.9	77.9	516 ± 140
Jul 29, 2019	Most	40 ± 30	794 ± 599	0.5	11.0	1,733 ± 1,308
Sep 30, 2019	Most	114 ± 46	2,403 ± 957	1.0	21.7	2,662 ± 1,060

*Numbers represent average ± standard error. Doubling times were calculated assuming two different *P*_*i*_ concentrations: *P*_*i*_ = *SRP* and *P*_*i*_ = 0.1 μg L^–1^. *SRP* values under detection limit were taken as 0.3 μg L^–1^. Note different doubling time units for epilithon (days) and seston (hours); PDT ratios were calculated as h/h.*

*SRP, soluble reactive phosphorus; *PDT*_*E*_, epilithon P doubling time; *PDT*_*sest*_, seston P doubling time.*

Abiotic ^33^P adsorption occurring in the presence of 4% formaldehyde was relatively high, accounting on average for 8% (range 1–18%), 24% (7–61%), and 54% (21–90%) of the formaldehyde-untreated samples in Lakes Milada, Most, and Medard, respectively (Figure 4B). In three additional experiments, we applied formaldehyde at a wide range of P concentrations (0.1–10 mg P L^–1^). In two of those, we observed a roughly two-fold increase in P adsorption with increasing P concentration, whereas no concentration dependence was found in the remaining experiment (Supplementary Figure 5). We discovered that more than one half of the adsorbed P (50–70%) could be readily released back to the dissolved phase during a 1-h treatment of the samples with an oxalacetate-based surface wash reagent (Supplementary Table 3), which indicates a weak and reversible abiotic P adsorption. No kinetic P uptake parameters reported throughout this paper have been corrected for abiotic P uptake (see section “Discussion”).

### Seston P Dynamics and Comparison With Epilithon

Turnover time of dissolved orthophosphate measured in the lake water with ^33^P ranged from 7 min to 3.6 h, indicating moderate (in spring) to extreme (in summer and autumn) P deficiency in all lakes (Supplementary Table 5). Seston P uptake kinetics followed the Michaelis–Menten model (Supplementary Figure 6), yielding *V*_*max*_ estimates ranging from 0.29 to 1.3 μg P L^–1^ h^–1^ and *K*_*S*_ from 0.4 to 6.1 μg P L^–1^ (Table 5). The specific P uptake affinities in seston significantly increased (*p* < 0.01, ANOVA) from 240–2,800 L g OM^–1^ h^–1^ in April to 6,200–23,900 L g OM^–1^ h^–1^ in June and October, thus being two to four orders of magnitude higher (Table 5) than the corresponding epilithon-specific values (0.08–3.1 L g OM^–1^ h^–1^, Table 3). Estimates of seston P doubling time (*PDT*_*sest*_) ranged from 0.5 to 51 h or from 11 to 740 h, assuming *P*_*i*_ concentration equal to *SRP* or to 0.1 μg L^–1^, respectively, with a generally decreasing seasonal trend opposite to seasonal changes in *PDT*_*E*_. Ratio of *PDT*_*E*_ to *PDT*_*sest*_ ranged from 86 to 516 in April, while it increased to 1,730–27,300 in July and October (Table 4).

**TABLE 5:** A summary of estimates of kinetic parameters (*V*_*max*_ and *K*_*S*_ of Michaelis–Menten kinetics and specific P uptake affinity, *SPUA*_*sest*_) of P uptake by seston in lake water in three post-mining lakes in the Czech Republic in 2019.

**Date**	**Lake**	**TT-PO_4_ (h)**	**Epilimnion P-deficiency**	***V*_*max*_ (μg P L^–1^ h^–1^)**	***K*_*S*_ (μg P L^–1^)**	***V_*m*__*ax*_*/*K*_*S*_ (h^–1^)**	***SPUA*_*sest*_ (L g OM^–1^ h^–1^)**	***SPUA* ratio (*SPUA*_*sest*_/*SPUA*_*E*_)**
Apr 15, 2019	Milada	3.6	Moderate	0.84 ± 0.11	2.16 ± 0.59	0.39 ± 0.07	240	264 ± 202
Jul 22, 2019	Milada	0.18	Extreme	5.44 ± 0.47	0.72 ± 0.12	7.60 ± 0.77	18,500	126,500 ± 62,360
Oct 14, 2019	Milada	0.16	Extreme	5.53 ± 0.32	0.74 ± 0.08	7.43 ± 0.53	8,600	67,890 ± 36,260
Apr 8, 2019	Medard	2.8	Moderate	0.29 ± 0.06	6.13 ± 2.49	0.05 ± 0.01	410	453 ± 157
Jul 8, 2019	Medard	0.83	Extreme	1.20 ± 0.22	0.57 ± 0.31	2.10 ± 0.89	11,100	20,400 ± 3,840
Oct 21, 2019	Medard	0.19	Extreme	4.39 ± 0.6	1.28 ± 0.29	3.42 ± 0.46	14,000	96,640 ± 71,360
Apr 29, 2019	Most	1.2	Moderate	0.76 ± 0.19	1.36 ± 0.78	0.56 ± 0.24	2,800	3,187 ± 598
Jul 29, 2019	Most	0.12	Extreme	11.4 ± 1.8	1.54 ± 0.37	7.38 ± 0.93	23,900	23,630 ± 13,300
Sep 30, 2019	Most	0.27	Extreme	1.51 ± 0.09	0.39 ± 0.05	3.87 ± 0.38	6,200	20,240 ± 11,060

*Numbers represent the best-fit estimate of a parameter ± standard error. For *SPUA*_*E*_ values, see Table 3.*

*TT-PO_4_, turnover time of dissolved orthophosphate; Epilimnion P-deficiency, epilimnion P limitation status (Istánovics et al., 1992); SPUA ratio, the ratio of specific P uptake affinities for seston and for epilithon—numbers represent average ± SD from four (2 × 2) ratios from two depths and two sampling sites.*

## Discussion

Data on periphyton P uptake kinetics in lentic systems are extremely scarce in the literature (Hwang et al., 1998; Scinto and Reddy, 2003; Wolfe and Lind, 2010). Our study represents the first report on P uptake kinetics by epilithon assemblages naturally growing on stones in temperate oligo- to mesotrophic lakes. The only comparable data are available from very different freshwater habitats (a subtropical lake and wetlands) (Hwang et al., 1998; Scinto and Reddy, 2003). The values of specific P uptake affinity (*SPUA*_*E*_) measured in this study (0.08–3.1 L g OM^–1^ h^–1^) were roughly one order of magnitude lower than those presented for periphyton by Scinto and Reddy in subtropical wetlands (Scinto and Reddy, 2003). While *V*_*max*_ estimates presented in our study (1.9–129 mg P g OM^–1^ h^–1^) roughly match the values available in the literature, our *K*_*S*_ estimates (3.9–135 mg P L^–1^) are one to three orders of magnitude higher than those previously reported (Hwang et al., 1998; Scinto and Reddy, 2003). The among-system differences in kinetic parameters might be attributed to the differences in abiotic and biotic drivers affecting P uptake in compared ecosystems, in the species composition of periphyton, environmental conditions, and morphological and limnological variances between studied lakes. In freshwater wetlands, extreme periphytic N:P ratios (152–231) have been reported (Scinto and Reddy, 2003). Such high values suggest a strong P limitation of these wetlands and may explain the 10 times higher *SPUA*_*E*_ compared with our estimates. A positive relationship between the degree of P deficiency and *SPUA*_*E*_ is well documented in algae (Krumhardt et al., 2013; Cáceres et al., 2019). The wide range of linear increase in P uptake velocity with a concentration of dissolved P, reflected by high *K*_*S*_ values or even by the absence of saturation, might represent an adaptation of the epilithon community to episodic increases of dissolved P, ensuring optimal utilisation of available resources.

Variability in *SPUA*_*E*_ was statistically related to both epilithon C:N molar ratio and lake-water pH. The negative relation between *SPUA*_*E*_ and epilithon C:N could indicate a tendency to a decrease in specific P uptake affinity under conditions of N+P co-limitation of the epilithic microbial community (Hillebrand and Sommer, 1999). Notably, the highest epilithon C:N ratios (approximately 22) were observed at Lake Milada in July and October (Figure 2C), concomitantly with low *SPUA*_*E*_ values (Figure 4A) and an extremely low lake-water nitrate concentration (0.02 mg L^–1^; Table 1), generally considered to be limiting for algal growth (Maberly et al., 2002). Since the naturally growing epilithon consist of a variety of species, some of them might have been N-limited, whereas the others might have been P-limited. Co-limitation of periphyton by N+P in oligotrophic lakes has been previously reported (Hogan et al., 2014). Following this logic, this could have led to a lower P demand and lower *SPUA*_*E*_ values of the epilithic community. However, the relation of *SPUA*_*E*_ to species composition was not a subject of this study. The positive relationship between *SPUA*_*E*_ and pH remains rather obscure, since the reported pH values (8–9) are common in lakes worldwide (Michelutti et al., 2002; Liess et al., 2009; Keshri et al., 2017). The effect was most probably indirect, as no clues revealing the direct effect of pH on P uptake traits have been reported in the literature yet.

In the lakes studied, epilithon biomass per area and nutrient stoichiometry corresponded with the values reported for comparable oligotrophic lakes (Liess et al., 2009). Two slightly different stoichiometric definitions of growth-limiting conditions were suggested in the literature. According to Hillebrand and Sommer, P limitation of benthic algae is indicated when C:P > 180 and N:P > 22 (Hillebrand and Sommer, 1999). Kahlert reported the threshold for P limitation of periphyton when C:P > 369 and N:P > 32 (Kahlert, 1998). Applying both definitions, the values found in the investigated post-mining lakes (average C:P 680 and N:P 39) indicate P limitation of epilithic growth, which tends to strengthen over the season (Figure 2B). Comparably high epilithic C:P and N:P ratios were also reported for deep oligotrophic lakes in Sweden (C:P and N:P ratios up to 800 and 74, respectively) (Liess et al., 2009) and an oligotrophic lake in north-western Ontario (C:P ratio 820) (Frost et al., 2002).

We observed a discrepancy between the seasonal development of *SPUA*_*E*_ and of the other limitation indices. Epilithon biomass cover (OM cm^–2^) roughly doubled, while biomass P content roughly halved from April to July and October. One would expect a simultaneous increase in *SPUA*_*E*_ in response to strengthened P limitation (Krumhardt et al., 2013; Cáceres et al., 2019) implied by an observed decrease in P content and increase in C:P ratio. However, the opposite was found; i.e., *SPUA*_*E*_ decreased roughly 10 times during the same period (Table 2 and Figure 4A). There are several possible explanations for this apparent contradiction: (1) the decreasing ratio of active living biomass to structural (mainly extracellular) polymers or detritus during the seasonal succession: *SPUA*_*E*_ was normalised to the total OM content, not discriminating living and dead cells as well as extracellular polymeric substances. This notion appears to be supported by the fact that the C:P ratio doubled from spring (∼410) to the extreme values in summer and autumn (∼820, Figure 2B). Moreover, the generally decreasing growth and activity of epilithon during vegetation season were proved in a parallel study (Čapková et al., in prep.) showing a roughly one-order-of-magnitude decrease in OM-normalised *in situ* primary production of epilithon in all lakes. (2) Seasonal changes in the epilithic microbial community might favour species with lower P requirements or motile growth form able to glide out from biofilm. Seasonal shifts in the phototrophic assemblage composition were in fact observed; however, it is statistically unrelated to *SPUA*_*E*_ (Bešta et al., in prep.). It has been reported that higher periphyton C:P ratios can be attributed to a higher proportion of heterotrophic to autotrophic organisms (Fitter and Hillebrand, 2009). (3) The high efficiency of P internal recycling in epilithon (ratio of P recycling to leakage into the lake water): phosphorus releasing out from the decomposing biomass may utilised by the living cells without leaving the biofilm and thus lowering the demand for external P input. (4) The decreased accessibility of P to epilithic microorganisms due to the thickening of the epilithic biofilm on the stone over the season: if true, this would markedly constrain P flux through the epilithon matrix described as a power function of flow velocity and as a negative power function of distance from the leading edge of the communities (Riber and Wetzel, 1987). As a result, the cells situated closer to the stone substrate could exhibit lower P uptake than the cells near the biofilm–water interface even if their individual P uptake affinities were high. Consequently, this would lead to lower values of overall P uptake and of the related parameters (*SPUA*_*E*_, *V*_*max*_, etc.) of the epilithon.

Values of epilithic specific P uptake affinity, *SPUA*_*E*_, were two to four orders of magnitude lower than the corresponding values for seston, *SPUA*_*sest*_, in all lakes studied (Table 4) with the highest difference (20,000–126,000-fold) in June and October. Similar but not as high (∼200-fold) differences in specific P uptake affinity were also reported for a shallow subtropical lake in Florida (Hwang et al., 1998). The differences between *SPUA*_*sest*_ and *SPUA*_*E*_ likely result from the mostly unicellular arrangement of sestonic bacterio- and phytoplankton that tends to maximise surface to volume ratio (Tambi et al., 2009), while epilithic P uptake is affected by multiple factors, e.g., a limited surface for nutrients entering the biofilm or the presence of an extracellular matrix interfering with nutrient transport from water into the cells (Riber and Wetzel, 1987; Larned et al., 2004).

Noteworthy, P uptake rate is calculated as a product of *SPUA* and the concentration of bioavailable P (*P*_*i*_) in the lake water. Assuming homogenous P concentration in the epilimnion, both planktonic and epiphytic organisms are exposed to the same P levels. Therefore, irrespectively to actual P concentration, planktonic microbes were able to acquire up to 20,000–126,000 times more P per unit of biomass than epilithic ones.

Likewise, the time necessary for doubling their P content was up to 1.7,000–27,000 times shorter for seston than for epilithon in summer and autumn (Table 4), implying extremely slow growth of epilithic compared with sestonic microorganisms.

Reliability of estimates of P uptake rates and P doubling times depend on knowledge of actual *P*_*i*_ concentrations. *SRP* is not a reliable estimate of the lake-water *P*_*i*_ concentration at its low levels (*SRP* < 5 μg P L^–1^), as it is determined with molybdenum blue method known to highly (up to 100 times) overestimate the actual *P*_*i*_ concentration under such conditions (Rigler, 1966) mainly due to the interference with arsenates and hydrolysed organic phosphorus (see, e.g., Tanaka et al., 2006). Very short values of turnover time of dissolved orthophosphate measured in epilimnia of all the studied lakes (7–50 min, Table 5) were shown to be associated with extremely low *P*_*i*_ concentrations (in nanomolar range, i.e., ∼0.1 μg L^–1^) (Rigler, 1966; Taylor and Lean, 1991; Chen and Taylor, 2011). In accordance, seston P doubling time values calculated in this study assuming *P*_*i*_ = 0.1 μg L^–1^ (i.e., 3.2 nmol L^–1^) resulted in summer and autumn in doubling times in tens of hours, which fit typical growth rates of sestonic microorganisms, while the assumption *P*_*i*_ = *SRP* (i.e., 9.6–102 nmol L^–1^) led to unrealistically short values (frequently approximately 1 h; see Table 4). Hence, doubling times calculated by *P*_*i*_ concentration 0.1 μg L^–1^ seem to be closer to the reality than the ones calculated by using *SRP*. In April, on the other hand, the seston doubling times calculated assuming *P*_*i*_ = *SRP* seemed to be realistic (Table 4), while those calculated assuming *P*_*i*_ = 0.1 μg L^–1^ were too long (∼80–500 h). This would imply that in April, when water column stratification and P deficiency in the lakes were not yet fully developed, *P*_*i*_ was of the order of micrograms per litre (∼10–100 mnol L^–1^) and close to *SRP*, while under conditions of severe P limitation occurring in July and October, it was roughly one order of magnitude lower (∼1–10 mnol L^–1^). Interestingly, estimates of residual *P*_*i*_ concentrations in P limited algal cultures matched the latter range (Laws et al., 2011; Grant et al., 2013). At any rate, epilithon P doubling times were always extremely long compared with seston (thousands of days vs. tens of hours; Table 4).

Consequently, epilithon microorganisms might not be able to acquire enough P for their growth when lakes are thermally stratified (from spring to autumn) and might be virtually outcompeted for P by planktonic microorganisms dominating P uptake in this period. This has important implications for the coexistence of plankton and periphyton, affecting biogeochemical cycles in the lakes studied and supposedly in deep oligotrophic lakes in general. Epilithon could prosper mostly in late autumn, winter, or early in the spring, when the mixing of the water column increases P availability in the upper strata (Wetzel, 2001) and phytoplankton productivity is limited (Reynolds, 2006). Such a relationship between plankton and epilithon may be interpreted as an example of temporal niche separation, allowing a stable and long-term coexistence of both communities in the same ecosystem (Chesson, 2000). Facility of epilithon to acquire P from the solid substrate should not be forgotten; however, concentration of P in the solid substrate at investigated lakes was negligible (Supplementary Table 4) compared with values available in the literature (Porder and Ramachandran, 2013). Therefore, we assume that it does not play an important role in the investigated lakes.

The use of naturally growing epilithon assemblage in the present study is challenging, but on the other hand, it provides more ecologically relevant data than commonly used artificial substrates (Jackson et al., 2001; Mieczan and Tarkowska-Kukuryk, 2012). An obvious disadvantage is an inherent heterogeneity of individual samples, resulting in a high scatter of P uptake data (see Supplementary Figures 2–4), which, however, does not rule out the general patterns and temporal trends to be identified.

Values of P uptake parameters reported throughout this paper have not been corrected for abiotic P uptake (i.e., uptake occurring in the presence of 4% formaldehyde), since the results of abiotic adsorption experiments were inconclusive, showing variable concentration dependency of the formaldehyde effect (Supplementary Figure 5), which does not allow P uptake to be corrected properly. However, when subtracting the proportion of the abiotic P uptake at the lowest *P*_*add*_ (the closest concentration to the lake-water concentration), its proportion increased with the season (Figure 4B). This would lead to even bigger seasonal decrease in *SPUA*_*E*_, providing stronger support for the main findings of this study. The abiotic P uptake was substantial, averaging at 54% of total P uptake in Lake Medard, 24% in Lake Most, and 8% in Lake Milada (Figure 4B).

In additional experiments, oxalacetate-based surface wash reagent released 50–70% of such abiotically adsorbed P, indicating a weak and reversible P binding (Supplementary Table 3). Similarly, Sañudo-Wilhelmy et al. (2004) reported surface abiotic P adsorption ranging from 60 to 90% of the apparent total P uptake in the phytoplankton. Surface P adsorption has been suggested to be a mechanism for storing the temporary excess of P before it can be transported into the algal cells (Yao et al., 2011). Exact mechanism of abiotic binding is not clarified, but it can depend on specific proteins (Scanlan et al., 1993; Kamennaya et al., 2020). Our data indicate that the phenomenon of temporary abiotic P adsorption could be important also in periphyton, but so far, this has not been considered and thus requires further attention in both theoretical and experimental studies.

## Data Availability Statement

The raw data supporting the conclusions of this article will be made available by the authors, without undue reservation.

## Author Contributions

EK and JN designed and performed the experiments, interpreted the results, and wrote the manuscript. KČ, PZ, MŘ, EK, JN, and KŘ performed the field work. PZ, PČ, KČ, KŘ, and MP reviewed the early version of the manuscript. PČ analyzed the data and ran the statistical analyses. KČ designed a map of lakes and took photos of epilithon. PZ took aerial photos of the lakes (presented in the Supplementary Material). All authors reviewed and approved the final version of the manuscript.

## Conflict of Interest

The authors declare that the research was conducted in the absence of any commercial or financial relationships that could be construed as a potential conflict of interest.

## Publisher’s Note

All claims expressed in this article are solely those of the authors and do not necessarily represent those of their affiliated organizations, or those of the publisher, the editors and the reviewers. Any product that may be evaluated in this article, or claim that may be made by its manufacturer, is not guaranteed or endorsed by the publisher.
