# Supplementary material for: Low Specific Phosphorus Uptake Affinity of Epilithon in Three Oligo- to Mesotrophic Post-mining Lakes
Source: Front Microbiol. 2021 Oct 6;12:735498. doi: 10.3389/fmicb.2021.735498 (PMC8527014; doi:10.3389/fmicb.2021.735498)
Supplement: Supplementary file 1 [file Data_Sheet_1.pdf]

Low specific P uptake affinity of epilithon in three oligo- to mesotrophic post-mining lakes
Supplementary Information

Eliška Konopáčová, Jiří Nedoma, Kateřina Čapková, Petr Čapek, Petr Znachor, Miloslav Pouzar, Milan Říha & Klára Řeháková

Supplementary Table 1 Results of Two-way ANOVA analysis of P uptake related epilithon variables measured in three post-mining lakes in the Czech Republic in 2019, with Lake and Sampling season as factors. Data with variability exceeding one order of magnitude were log-tranformed.

	**Lake**			**Sampling season**			**Interaction**		
**Epilithon variable**	**% of var. expl.**	**F (DFn, DFd)**	**p**	**% of var. expl.**	**F (DFn, DFd)**	**p**	**% of var. expl.**	**F (DFn, DFd)**	**p**
**V_{max} (mgP gOM^{-1} h^{-1})**	1.83	$F_{2,19} = 0.087$	0.439	50.9	$F_{2,19} = 24.28$	**<0.0001**	5.70	$F_{4,19} = 1.359$	0.2975
**K_S (mgP L^{-1})**	0.17	$F_{2,19} = 0.042$	0.9593	14.3	$F_{2,19} = 3.58$	0.0552	29.15	$F_{4,19} = 3.665$	**0.0304**
**Specific P uptake affinity (L gOM^{-1} h^{-1})**	13.09	$F_{2,26} = 6.510$	**0.0051**	47.64	$F_{2,26} = 23.69$	**<0.0001**	14.47	$F_{4,26} = 3.600$	**0.0183**
**Biomass (mgOM cm^{-2})**	47.83	$F_{2,26} = 23.61$	**<0.0001**	13.76	$F_{2,26} = 6.791$	**0.0042**	11.13	$F_{4,26} = 2.746$	**0.0498**
**Biomass (mgDW cm^{-2})**	46.59	$F_{2,26} = 20.84$	**<0.0001**	15.71	$F_{2,26} = 7.028$	**0.0036**	9.17	$F_{4,26} = 2.05$	0.1166
**OM/DW (%)**	44.66	$F_{2,26} = 13.33$	**0.0001**	1.95	$F_{2,26} = 0.580$	0.5668	8.22	$F_{2,26} = 1.226$	**0.3241**
**P content (mgP gOM^{-1})**	10.32	$F_{2,26} = 3.183$	0.058	42.85	$F_{2,26} = 13.22$	**0.0001**	3.58	$F_{4,26} = 0.553$	0.6989
**P content (µgP cm^{-2})**	52.24	$F_{2,26} = 17.68$	**<0.0001**	1.91	$F_{2,26} = 0.648$	0.5314	7.35	$F_{4,26} = 1.244$	0.3171
**C:P (molar ratio)**	30.36	$F_{2,26} = 13.1$	**0.0001**	36.16	$F_{2,26} = 15.61$	**<0.0001**	4.11	$F_{4,26} = 0.887$	0.4857
**C:N (molar ratio)**	41.53	$F_{2,26} = 45.93$	**<0.0001**	43.74	$F_{2,26} = 48.37$	**<0.0001**	9.62	$F_{4,26} = 5.322$	**0.0029**
**N:P (molar ratio)**	22.21	$F_{2,26} = 5.747$	**0.0086**	22.98	$F_{2,26} = 5.947$	**0.0075**	3.65	$F_{4,26} = 0.472$	0.7557

% of var. expl., per cent of total variability explained.

Supplementary Figure 1 An example of time courses of ^{33}P orthophosphate uptake by epilithon in the sample from Lake Milada, 22 Jul 2019. Data show the disappearance of ^{33}P from incubation medium supplemented with different concentrations of unlabelled orthophosphate (P_{add}), curves are best fits to the second-order polynomial equation (**Eq. 1**).

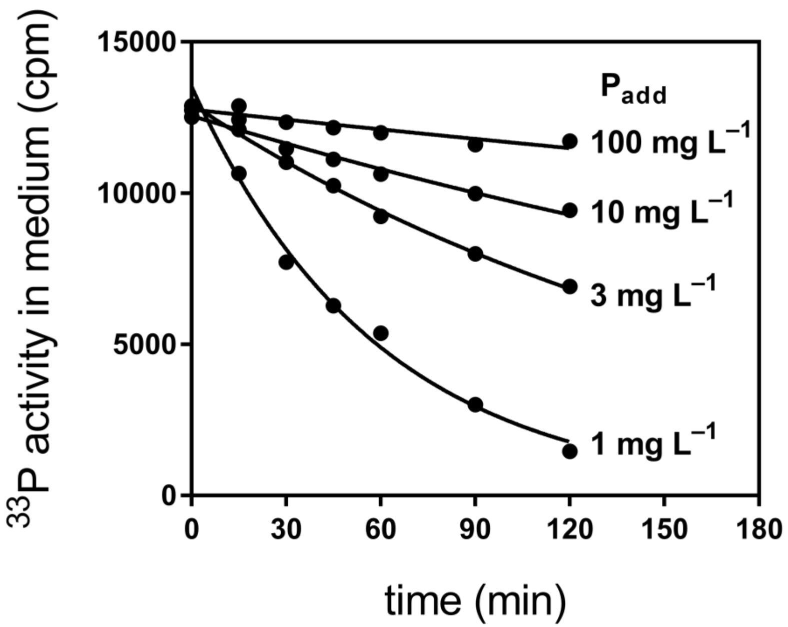

Supplementary Table 2 A summary of estimates of rate constants of P uptake (k_{upt}) by epilithon at different concentrations of unlabelled orthophosphate (P_{add}) in three post-mining lakes in the Czech Republic in 2019 with indices of the goodness of fit. Each line represents four experiments (2 depths, two sites) in duplicates. P_{add}, concentration of added orthophosphate.

**Lake**	**Month**	**P_{add} (mgP L^{-1})**	**k_{upt} (h^{-1}) average (range)**	**k_{upt} standard error (%) average (range)**	**R^2adj average (range)**
Milada	Apr	1	0.372 (0.164—0.761)	7 (3.2—14.5)	0.942 (0.788—0.991)
Milada	Apr	3	0.627 (0.216—1.77)	7.2 (0—14.1)	0.948 (0.833—1)
Milada	Apr	10	0.379 (0.18—0.827)	9.2 (3.9—19.9)	0.932 (0.804—0.997)
Milada	Apr	30	0.216 (0.125—0.35)	6.6 (1.4—12.6)	0.941 (0.849—0.997)
Milada	Apr	100	0.167 (0.074—0.364)	11.8 (1.7—26.3)	0.858 (0.576—0.997)
Milada	July	1	0.502 (0.196—1.184)	10.5 (0.3—27.5)	0.912 (0.76—1)
Milada	July	3	0.252 (0.06—0.386)	6.9 (3.5—16.3)	0.953 (0.809—0.999)
Milada	July	10	0.14 (0.064—0.322)	13.4 (3.9—23.1)	0.868 (0.688—0.999)
Milada	July	30	0.051 (0.025—0.088)	11.7 (3.5—24.1)	0.896 (0.684—0.993)
Milada	July	100	0.049 (0.019—0.082)	15.5 (3.1—25.2)	0.848 (0.668—0.997)
Milada	Oct	1	0.185 (0.075—0.443)	7.6 (1.4—23.7)	0.941 (0.715—0.999)
Milada	Oct	3	0.141 (0.053—0.24)	8.4 (3.3—15.5)	0.942 (0.871—0.999)
Milada	Oct	10	0.108 (0.042—0.413)	11.7 (3.3—39.4)	0.899 (0.478—0.995)
Milada	Oct	30	0.046 (0.02—0.083)	13.8 (5—22.8)	0.884 (0.761—0.983)
Milada	Oct	100	0.046 (0.014—0.085)	13.7 (1—23.1)	0.873 (0.723—1)
Medard	Apr	1	0.256 (0.143—0.455)	8 (2.4—11.5)	0.961 (0.9—0.992)
Medard	Apr	3	0.225 (0.117—0.811)	4.1 (1.7—7.6)	0.977 (0.942—0.997)
Medard	Apr	10	0.123 (0.076—0.224)	6 (3.4—9.4)	0.947 (0.888—0.998)
Medard	Apr	30	0.108 (0.074—0.202)	7.7 (3.9—13.8)	0.925 (0.799—0.995)
Medard	Apr	100	0.079 (0.045—0.122)	10 (3.5—23.1)	0.875 (0.602—0.987)

P_{add}, concentration of added orthophosphate.

Supplementary Table 2 *(cont.)*

Lake	Month	P_{add} (mgP L^{-1})	k_{upt} (h^{-1}) average (range)	k_{upt} standard error (%) average (range)	R^2adj average (range)
Medard	July	1	0.265 (0.104—0.538)	8.2 (4—15.3)	0.95 (0.853—0.994)
Medard	July	3	0.21 (0.101—0.371)	6.7 (3.1—11.2)	0.959 (0.898—0.992)
Medard	July	10	0.142 (0.063—0.246)	6.7 (1.1—14.8)	0.954 (0.859—0.999)
Medard	July	30	0.071 (0.014—0.149)	12.8 (4.5—32.6)	0.877 (0.534—0.981)
Medard	July	100	0.045 (0.006—0.102)	15.4 (0—35.2)	0.68 (-0.188—0.981)
Medard	Oct	1	0.152 (0.08—0.326)	13.2 (1.3—36.8)	0.839 (0.456—0.999)
Medard	Oct	3	0.122 (0.063—0.288)	14 (6.6—28.8)	0.849 (0.544—0.987)
Medard	Oct	10	0.07 (0.02—0.135)	17.2 (9.1—29.7)	0.81 (0.657—0.934)
Medard	Oct	30	0.048 (0—0.101)	13.3 (0—25.6)	0.719 (-0.2—0.968)
Medard	Oct	100	0.021 (0.01—0.027)	45.6 (27.3—93.2)	0.447 (0.019—0.747)
Most	Apr	1	0.326 (0.193—0.589)	7.9 (0.7—12.6)	0.943 (0.845—1)
Most	Apr	3	0.269 (0.132—0.503)	6.6 (2.7—13.6)	0.965 (0.895—0.993)
Most	Apr	10	0.286 (0.133—0.88)	4.7 (0.1—12.8)	0.958 (0.834—1)
Most	Apr	30	0.191 (0.103—0.401)	9.8 (0.5—18.1)	0.904 (0.797—1)
Most	Apr	100	0.102 (0.062—0.163)	11.7 (4.8—33.4)	0.884 (0.525—0.982)
Most	July	1	0.393 (0.142—0.716)	3.9 (1—9.6)	0.988 (0.963—1)
Most	July	3	0.312 (0.117—0.658)	5.9 (0.7—13.4)	0.977 (0.936—1)
Most	July	10	0.178 (0.087—0.287)	4 (0.7—9.4)	0.987 (0.945—1)
Most	July	30	0.059 (0.039—0.072)	10.1 (4.7—18.5)	0.922 (0.797—0.984)
Most	July	100	0.029 (0.016—0.043)	19.8 (3.7—43.3)	0.776 (0.373—0.994)
Most	Oct	1	0.171 (0.098—0.331)	4.1 (1.2—7.2)	0.983 (0.947—0.999)
Most	Oct	3	0.123 (0.081—0.187)	6.5 (2.2—14.5)	0.957 (0.856—0.997)
Most	Oct	10	0.075 (0.046—0.119)	6.4 (3.5—11.1)	0.967 (0.907—0.993)
Most	Oct	30	0.045 (0.017—0.094)	19.3 (3.8—36.8)	0.773 (0.46—0.991)
Most	Oct	100	0.021 (0.005—0.054)	33.3 (4.3—98.6)	0.636 (0.002—0.998)

P_{add}, concentration of added orthophosphate.

Supplementary Figure 2 A complete set of saturation data measured in experiments with P uptake by epilithon sampled at Lake Milada in 2019. L. Milada N and L. Milada S denote north and south sampling sites, respectively. Black symbols and curves show the best fits of either linear or Michaelis-Menten model, most parsimonious model was selected with F-test (see the main-text **Table 3** for details). Red points are outliers. Blue curves show fits based on integrated Michaelis Menten kinetics.

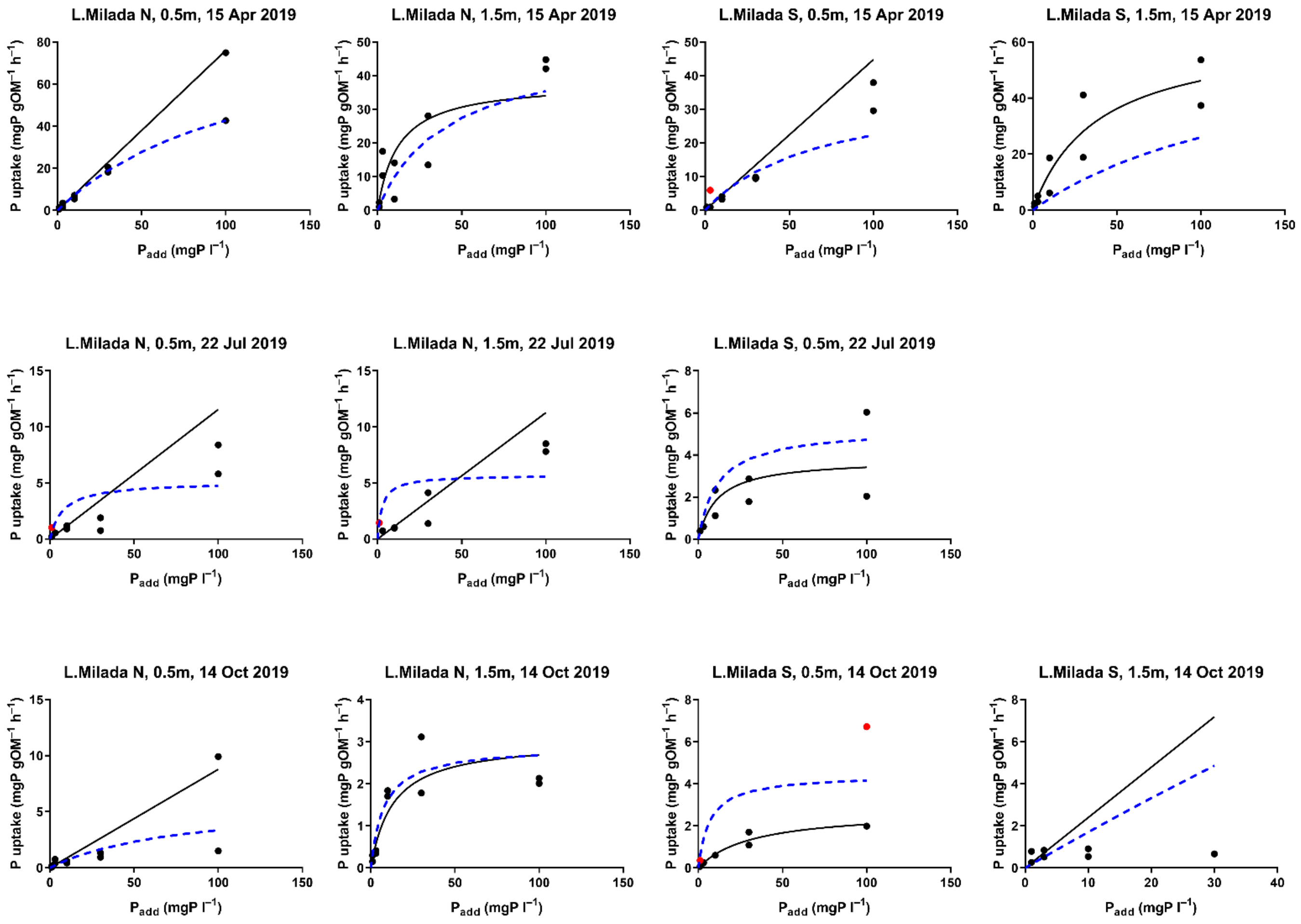

Supplementary Figure 3 A complete set of saturation data measured in experiments with P uptake by epilithon sampled at Lake Medard in 2019. L. Medard N and L. Medard S denote north and south sampling sites, respectively. Black symbols and curves show the best fits of either linear or Michaelis-Menten model, most parsimonious model was selected with F-test (see the main-text **Table 3** for details). Red points are outliers. Blue curves show fits based on integrated Michaelis Menten kinetics.

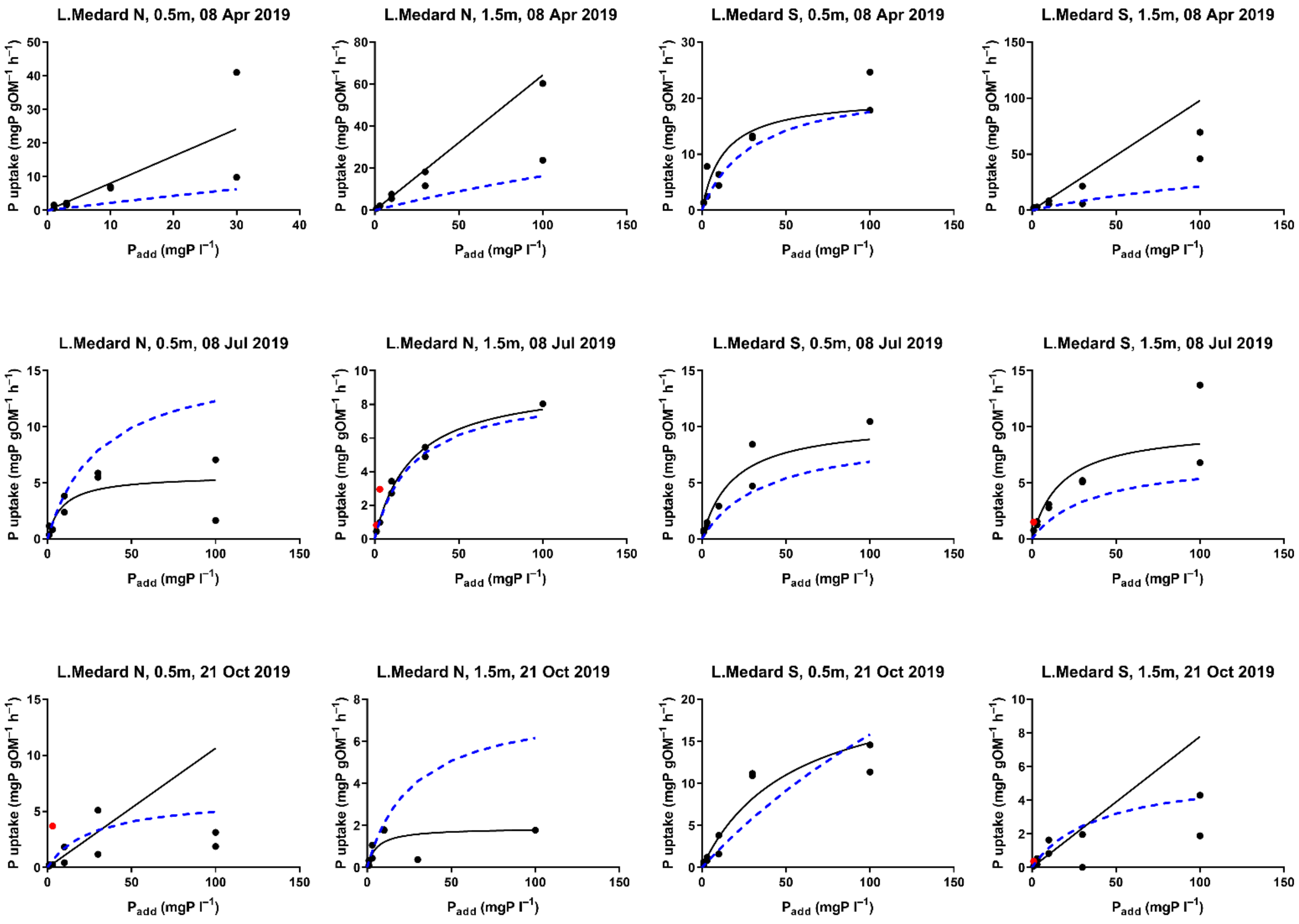

Supplementary Figure 4 A complete set of saturation data measured in experiments with P uptake by epilithon sampled at Lake Most in 2019. L. Most N and L. Most S denote north and south sampling sites, respectively. Black symbols and curves show the best fits of either linear or Michaelis-Menten models, most parsimonious model was selected with F-test (see the main-text **Table 3** for details). Red points are outliers. Blue curves show fits based on integrated Michaelis Menten kinetics.

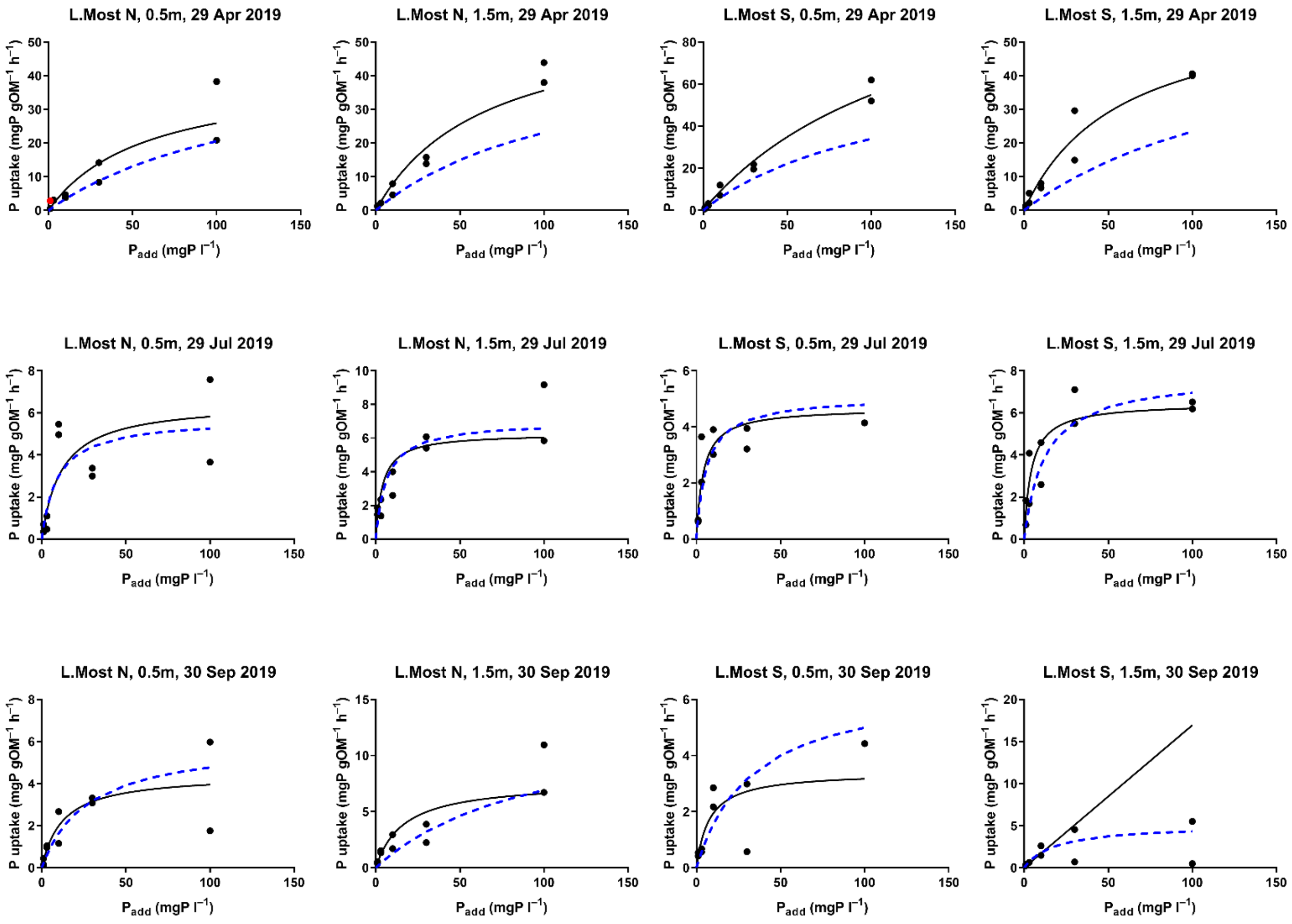

Supplementary Figure 5 Concentration dependence of the effect of 4% formaldehyde on P uptake by epilithon in three post-mining lakes in the Czech Republic in 2020. Data and curves show P uptake in the absence (tot - total uptake) or presence (abio - abiotic uptake or absorption) of formaldehyde and their difference (bio - biotic uptake). Red symbols show the percentage of abiotic uptake relatively to total one.

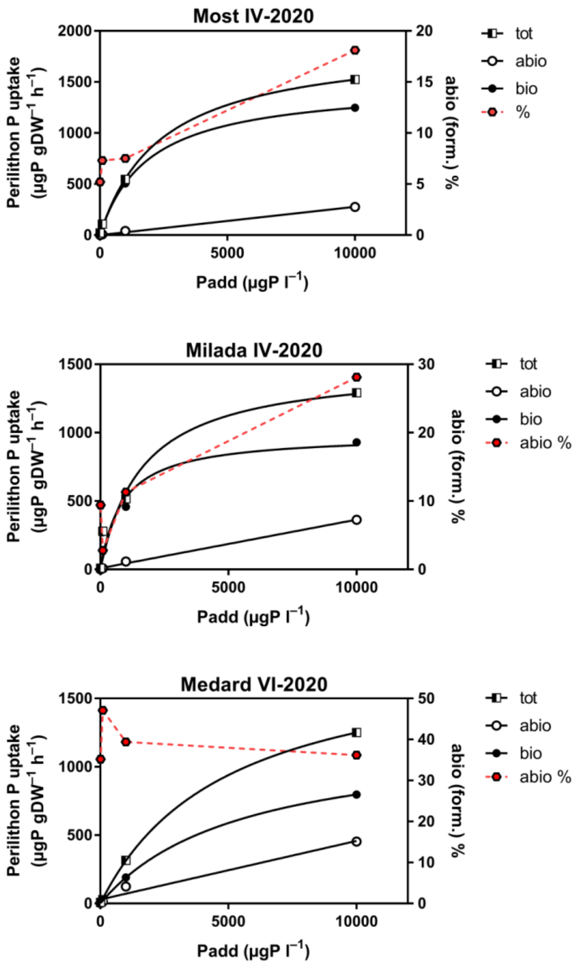

Supplementary Table 3 Percentages of abiotic P uptake (occurring in the presence of 4% formaldehyde) by epilithon from post-mining lakes in the Czech Republic, that could be released during one-hour incubation of with surface wash reagent (18.6 g L^{-1} $Na_2EDTA \times 2H_2O$, 14.7 g L^{-1} $C_6H_5Na_3O_7 \times 2H_2O$, 0.74 g L^{-1} KCl, 12.6 g L^{-1} $C_2H_2O_4 \times 2H_2O$, pH 8.0, Sanchez et al. 2003).

**Sample, date**	**Abiotic P uptake (% of control)**	**P release (% of Abiotic P uptake)**
Lake Milada, April 2020	9.4	69 (48–89), n=3
Lake Milada, June 2020	16.9	54 (42–67), n=4
Lake Medard, July 2020	73	50 (41–60), n=3
Lake Most, June 2020	8.9	52 (44–66), n=2

Supplementary Figure 6 A complete set of saturation data measured in experiments with P uptake by seston in lakewater samples from three postmining lakes sampled in the Czech Republic in 2019. Curves show the best fits with the Michaelis-Menten model. See the **main text Table 4** for details).

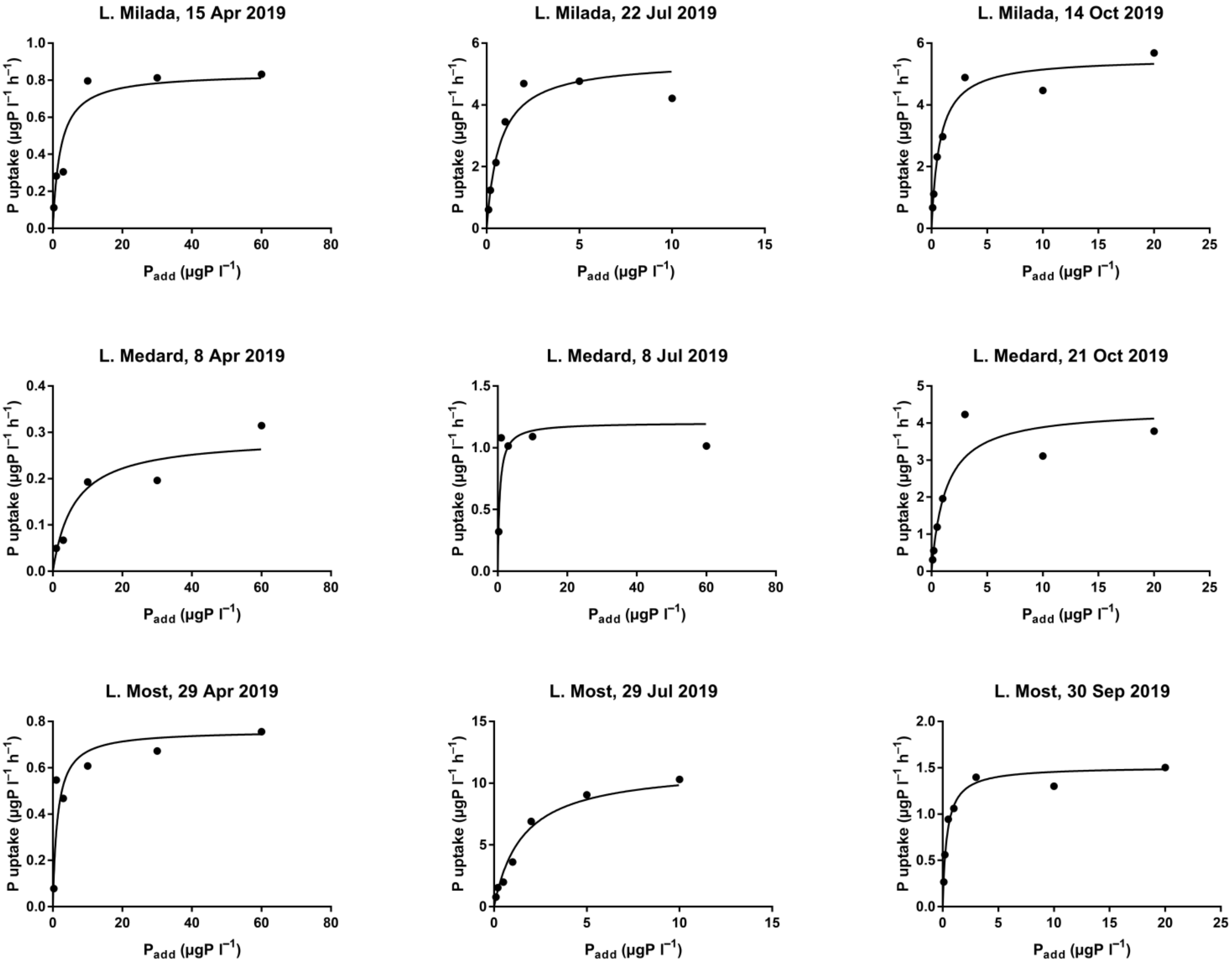

Supplementary Table 4 Characterisation of sampling sites at three post-mining lakes in the Czech Republic sampled in 2019.

Lake	Sampling site	GPS	Geology of stones	P in stones average ± st. error (ppm)
Milada	north shore	50°39'29.9"N 13°56'46.5"E	Potassium feldspar, quartz	3.28 ± 0.02
	south shore	50°39'08.5"N 13°56'14.0"E		
	lake central	50°39'21.1"N 13°56'29.3"E		
Medard	north shore	50°11'08.8"N 12°36'05.0"E	Granitoid – granite, biotit, cinvaldit, potassium feldspar, quartz	0.79 ± 0.01
	south shore	50°10'38.2"N 12°36'55.4"E		
	lake central	50°10'51.5"N 12°36'15.0"E		
Most	north shore	50°32'38.0"N 13°39'36.3"E	Basalt	3.29 ± 0.01
	south shore	50°32'06.5"N 13°38'06.9"E		
	lake central	50°32'42.7"N, 13°38'46.0"E		

Supplementary Figure 7 Lake Milada, aerial view from south-east. Photo P. Znachor.

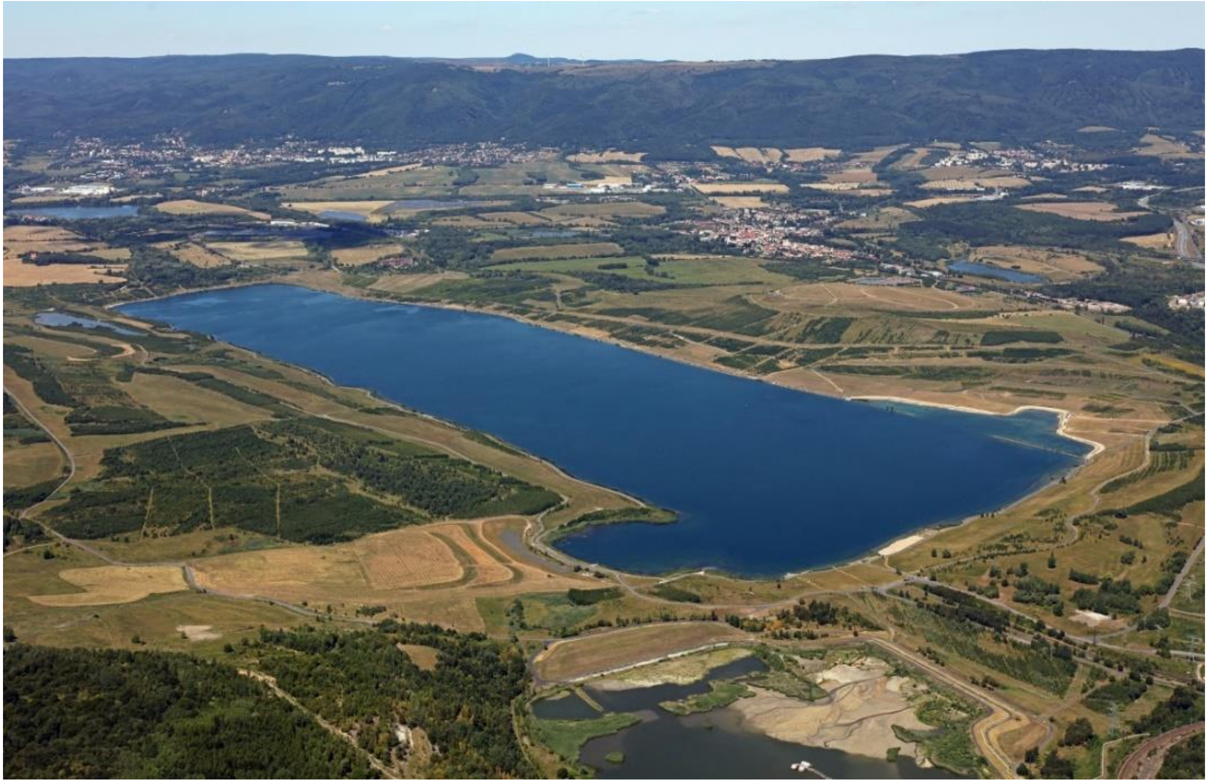

Supplementary Figure 8 Lake Medard, aerial view from north-west. Photo P. Znachor.

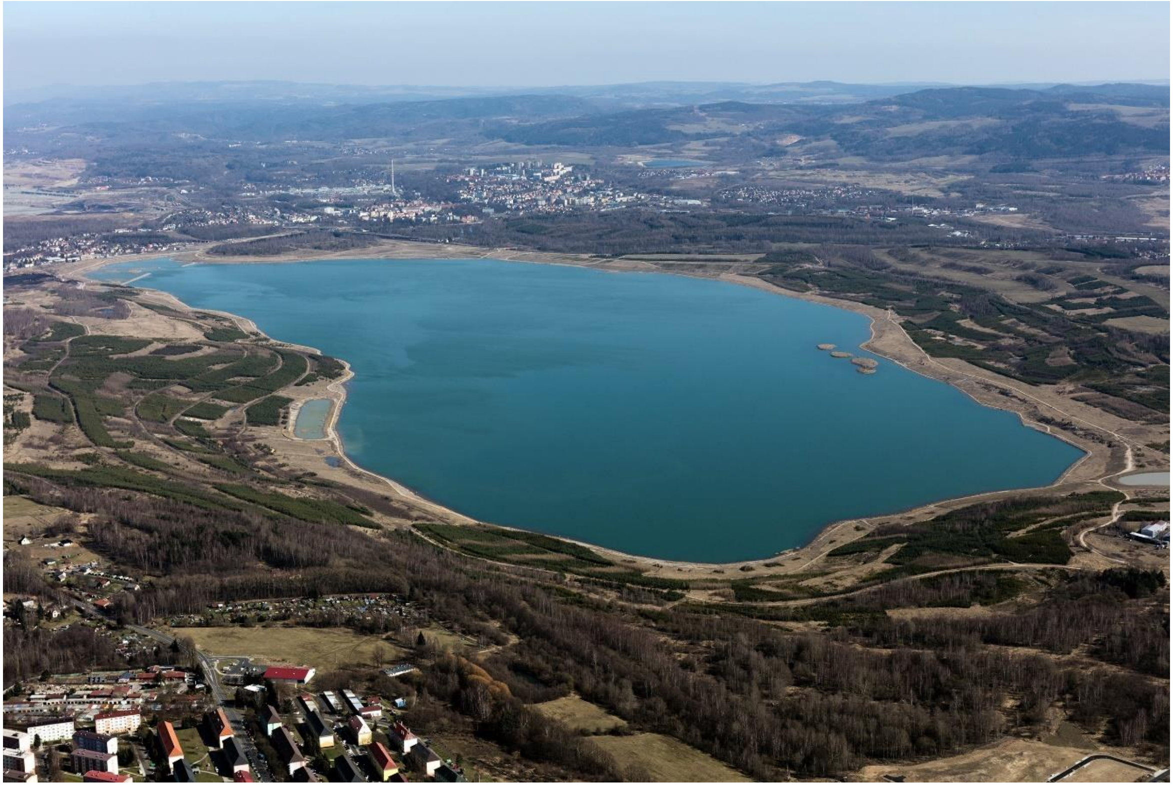

Supplementary Figure 9 Lake Most, aerial view from south. Photo P. Znachor.

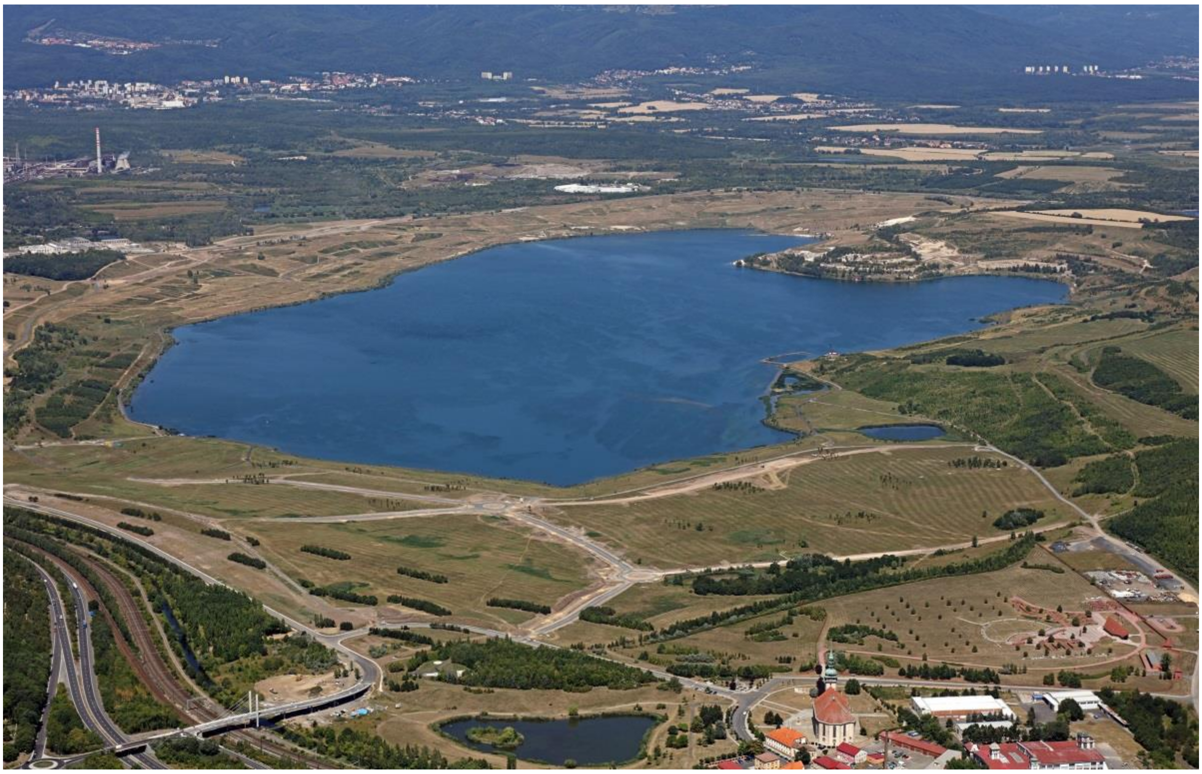

Supplementary Figure 10 Well-developed epilithon community in the litoral zone of Lake Milada, October 2019. Photo K. Čapková.

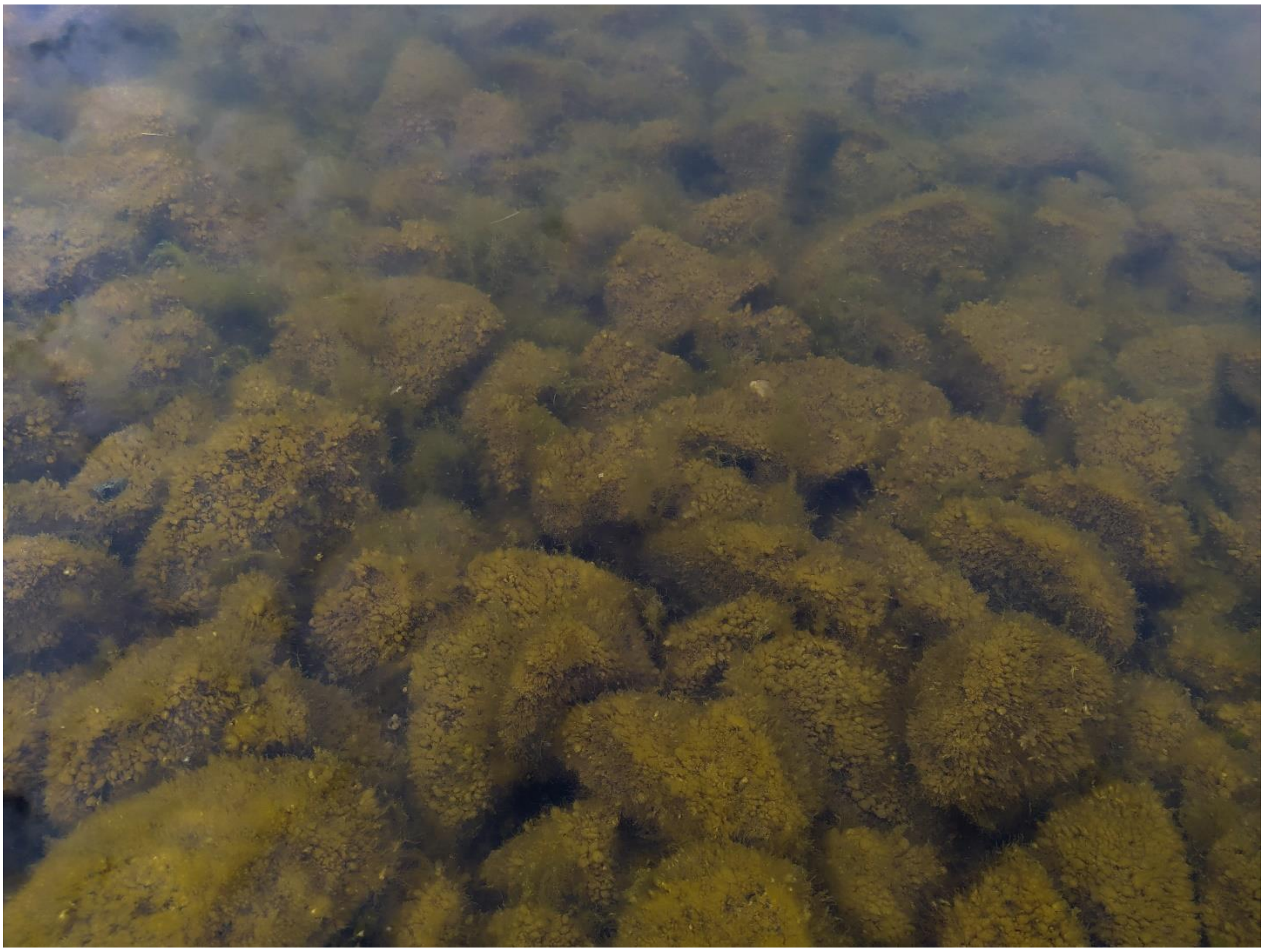

Estimation of P uptake kinetic parameters using a global fit using integrated form of Michaelis-Menten equation

We used an alternative approach of kinetic parameters (V_{MAX} and K_S) estimation. The integrated form of Michaelis-Menten equation represented by following equation:

$$t = -\frac{1}{V_{MAX}}\left[K_S \times ln\left(\frac{P(t)}{P_0}\right) + P(t) - P_0\right] \quad \text{(Suplementary eq. 1)}$$

was fitted to a data across all concentrations of added P. In this equation, t is time (in hours), $P(t)$ and P_0 are P concentrations at time t and zero (i.e. initial P concentration in mg(P) g(OM)$^{-1}$), respectively. The integrated form of Michaelis-Menten equation doesn't allow for the expression of P loss from the solution over time as a function of V_{MAX} and K_S directly. It allows to match the measured concentration of P (i.e. $P(t)$) to a time point at which such a concentration should theoretically appear based on respective P_0, V_{MAX} and K_S. The equation parameters V_{MAX} and K_S were estimated using the Differential Evolution algorithm (Mullen et al., 2011) minimizing the objective function J. The objective function J was defined as:

$$J = \sum_{i=1}^{n}(P_i - O_i)^2 \quad \text{(Suplementary eq. 2)}$$

where O_i and P_i stand for observation i and its corresponding value predicted by the integrated equation. Uncertainty of parameters estimates were additionally determined, using the parameter values defined by the Differential Evolution algorithm above as initial starting point, by Constrained Markov Chain Monte Carlo simulation on 10000 iterations using R package FME (Soetaert and Petzoldt, 2010).

References:

Mullen K, Ardia D, Gil D, et al (2011) “DEoptim”: An R Package for Global Optimization by Differential Evolution. J Stat Softw 40:1–26

Soetaert K, Petzoldt T (2010) Inverse Modelling, Sensitivity and Monte Carlo Analysis in R Using Package FME. J Stat Softw 33:1–28

Supplementary Figure 11 Residual P concentration in the solution over time. Different boxes represent different initial concentrations of P_{add}. The circles represent measured values and the black solid line represents the fit of integrated form of Michaelis-Menten equation (see Suplementary eq. 1) across all initial P concentrations. Lake Milada, North 0.5m, April

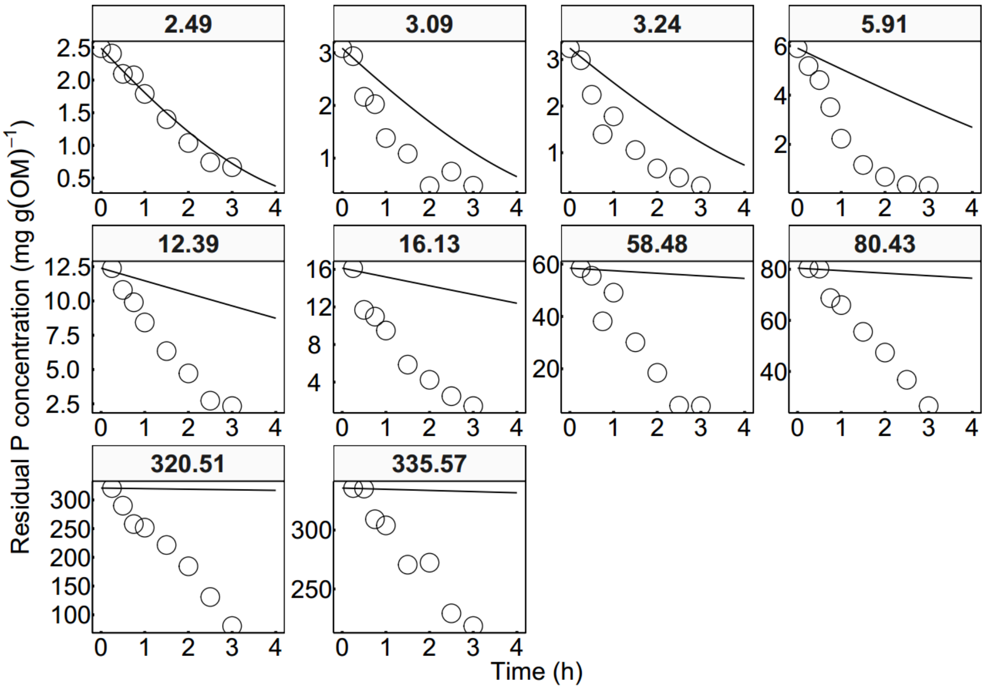

Supplementary Figure 12 Residual P concentration in the solution over time. Different boxes represent different initial concentrations of added P. The circles represent measured values and the black solid line represents the fit of integrated form of Michaelis-Menten equation (see Suplementary eq. 1) across all initial P concentrations. Sample Lake Milada, North 1.5m, July

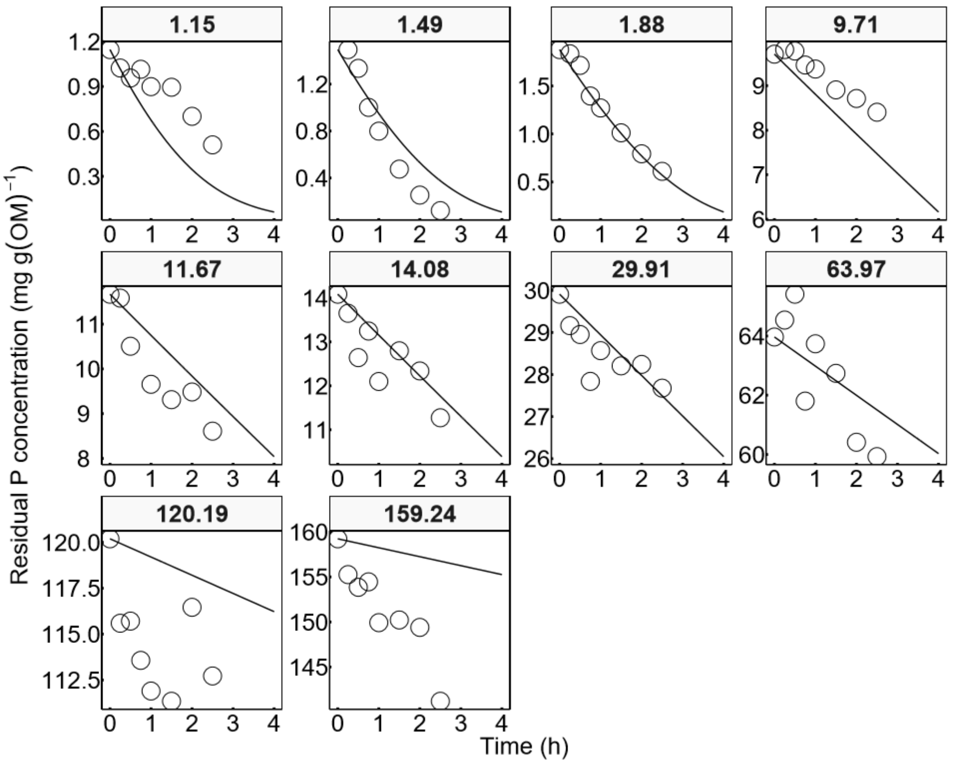

Supplementary Figure 13 Residual P concentration in the solution over time. Different boxes represent different initial concentrations of added P. The circles represent measured values and the black solid line represents the fit of integrated form of Michaelis-Menten equation (see Suplementary eq. 1) across all initial P concentrations. Lake Milada, North 1.5m, October

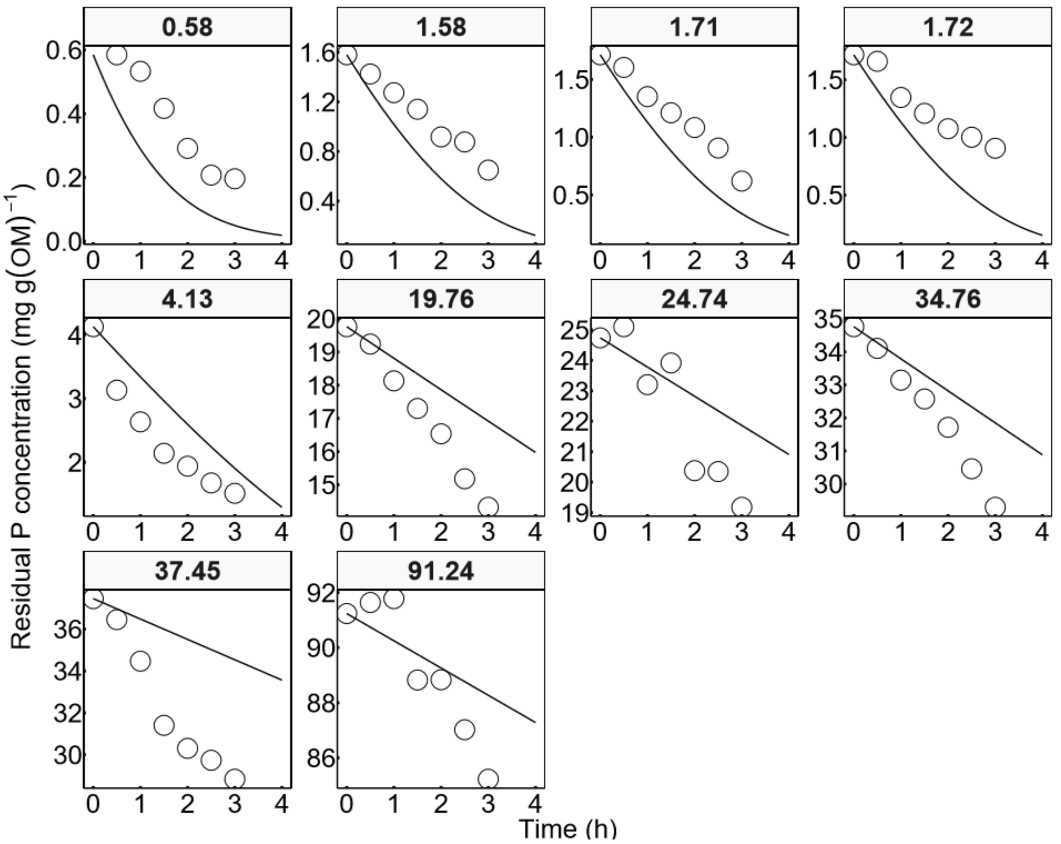

Supplementary Figure 14 A comparison of estimates of epilithon specific P uptake affinity ($SPUA_E$) calculated either using (a) global fit based on integrated Michaelis Menten kinetics (b) "traditional fit" based on initial uptake rate estimation followed by fit to Michaelis Menten model.

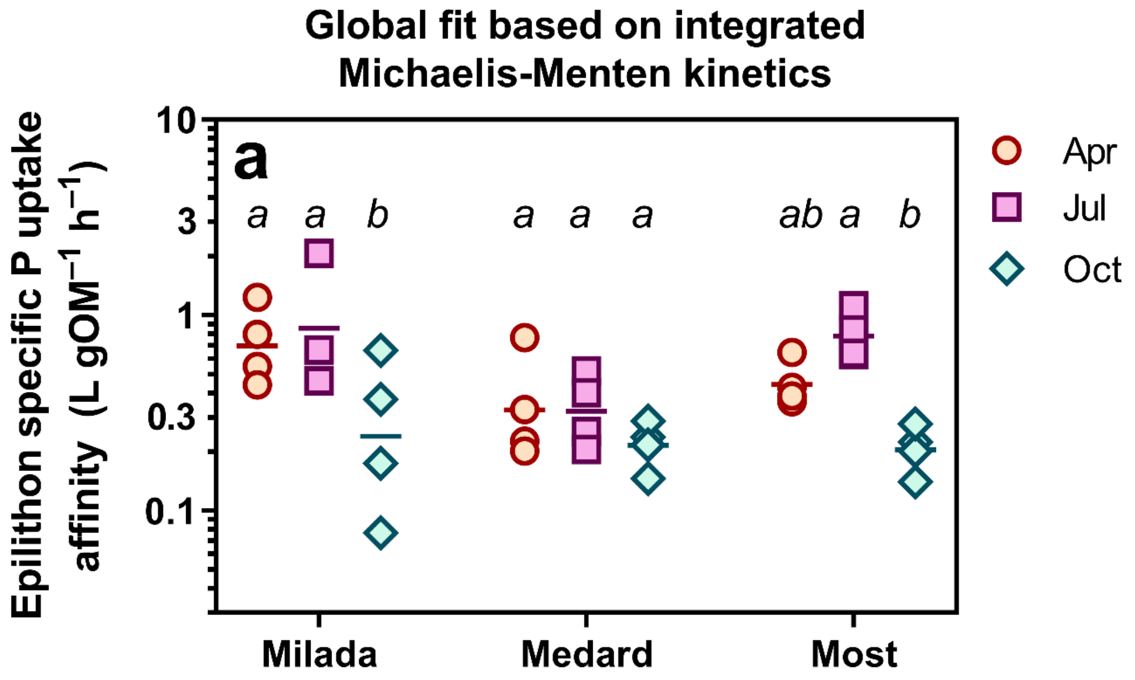

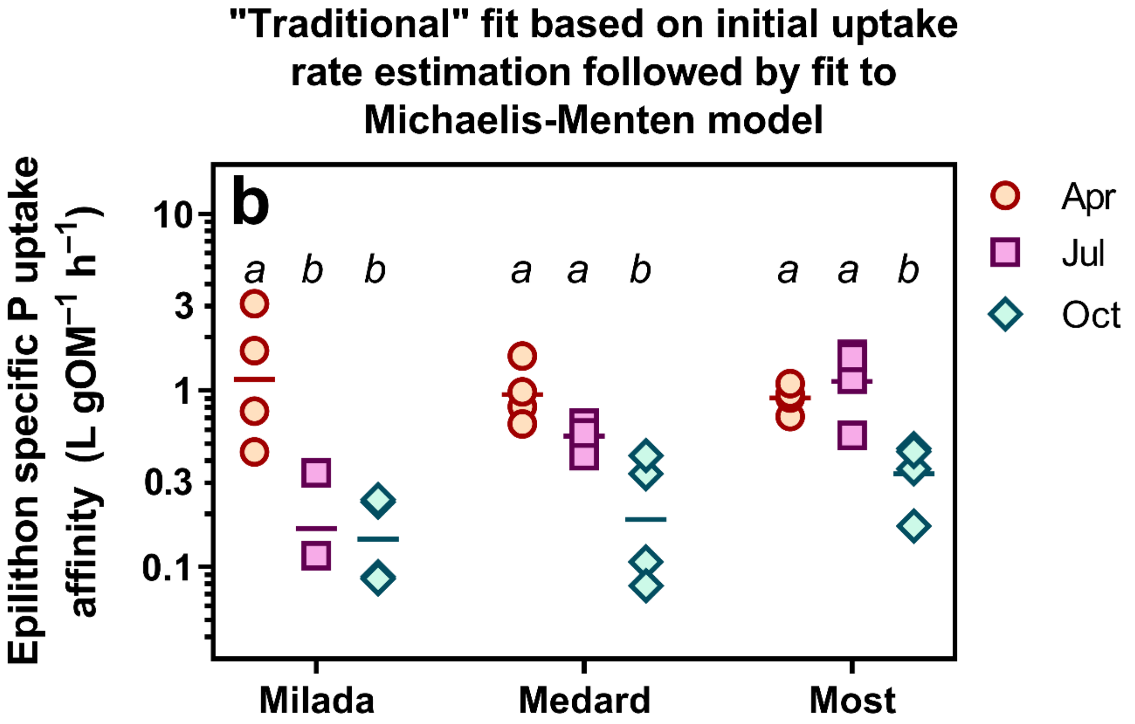

Supplementary Table 5 A comparison of estimates of parameters (± standard error) of Michaelis Menten model determined either using (i) global fit to integrated Michaelis Menten kinetics ($*V_{max}$, $*K_S$, $*SPUA_E$) or (ii) initial uptake rate estimation followed by fit to Michaelis Menten model (V_{max}, K_S, $SPUA_E$).

				**Estimates based on global fit based on integrated MM kinetics**			**Estimats based on initial uptake rate estimation followed by MM kinetics**			**$*SPUA_E$/ $SPUA_E$**
**Date**	**Lake**	**Site**	**Depth**	**$*V_{max}$** (mgP gOM^{-1} h^{-1})	**$*K_S$** (mg P L^{-1})	**$*SPUA_E$** (L gOM^{-1} h^{-1})	**V_{max}** (mgP gOM^{-1} h^{-1})	**K_S** (mg P L^{-1})	**$SPUA_E$** (L gOM^{-1} h^{-1})	
15-Apr-19	Milada	N	0.5	92.3 ± 11.6	116 ± 19	0.796	–	–	0.76 ± 0.099	1.05
15-Apr-19	Milada	N	1.5	49.7 ± 11.9	40.4 ± 13	1.23	38.2 ± 16.1	12.3 ± 8.4	3.101 ± 1.178	0.40
15-Apr-19	Milada	S	0.5	37.8 ± 11.4	69.0 ± 26.2	0.547	–	–	0.448 ± 0.075	1.22
15-Apr-19	Milada	S	1.5	63.6 ± 9.2	145 ± 28	0.440	63.8 ± 23.1	38.1 ± 18.4	1.674 ± 0.316	0.26
22-Jul-19	Milada	N	0.5	5.1 ± 1.2	7.7 ± 2.7	0.662	–	–	0.116 ± 0.023	5.73
22-Jul-19	Milada	N	1.5	5.7 ± 1.9	2.8 ± 1.6	2.064	–	–	0.113 ± 0.024	18.33
22-Jul-19	Milada	S	0.5	5.3 ± 1.2	11.4 ± 3.4	0.462	3.8 ± 1	11.2 ± 4.8	0.34 ± 0.083	1.36
14-Oct-19	Milada	N	0.5	5.9 ± 2.6	77.3 ± 40.3	0.077	–	–	0.088 ± 0.023	0.88
14-Oct-19	Milada	N	1.5	2.9 ± 0.6	7.8 ± 2.2	0.370	3 ± 0.7	13.1 ± 5	0.232 ± 0.048	1.60
14-Oct-19	Milada	S	0.5	4.4 ± 0.8	6.7 ± 1.6	0.660	2.7 ± 0.5	32 ± 7.2	0.085 ± 0.007	7.72
14-Oct-19	Milada	S	1.5	67.2 ± 13.8	384 ± 86	0.175	–	–	0.239 ± 0.099	0.73
08-Apr-19	Medard	N	0.5	94.1 ± 17.6	416 ± 90	0.226	–	–	0.806 ± 0.153	0.28
08-Apr-19	Medard	N	1.5	86 ± 16.3	427± 104	0.201	–	–	0.643 ± 0.073	0.31
08-Apr-19	Medard	S	0.5	22.7 ± 2.0	29.7 ± 4.3	0.766	20.3 ± 6.1	12.9 ± 6.2	1.567 ± 0.412	0.49
08-Apr-19	Medard	S	1.5	60.3 ± 8.3	184 ± 35	0.328	–	–	0.979 ± 0.21	0.34
08-Jul-19	Medard	N	0.5	16.1 ± 4.1	31.2 ± 9.8	0.517	5.7 ± 1.8	8.7 ± 4.9	0.651 ± 0.219	0.79
08-Jul-19	Medard	N	1.5	8.9 ± 4.4	22.4 ± 15.5	0.400	9.4 ± 1.1	22 ± 3.9	0.427 ± 0.035	0.94
08-Jul-19	Medard	S	0.5	9.5 ± 2.0	37.3 ± 10.4	0.254	–	–	0.574 ± 0.086	0.44
08-Jul-19	Medard	S	1.5	7.3 ± 0.9	35.3 ± 6.8	0.205	10 ± 2.4	17.5 ± 6.6	0.569 ± 0.115	0.36
21-Oct-19	Medard	N	0.5	6.3 ± 1.2	26.4 ± 6.9	0.238	–	–	0.106 ± 0.027	2.24
21-Oct-19	Medard	N	1.5	7.8 ± 2.5	27.3 ± 9.5	0.287	1.9 ± 0.8	5.6 ± 4	0.336 ± 0.139	0.85

Supplementary Table 5 (cont.)

				Estimates based on global fit to integrated MM kinetics			Estimats based on initial uptake rate estimation			***SPUA$_E$/ SPUA$_E$***
**Date**	**Lake**	**Site**	**Depth**	***V_{max}*** (mgP gOM^{-1} h^{-1})	***K_S*** (mg P L^{-1})	***SPUA$_E$*** (L gOM^{-1} h^{-1})	***V_{max}*** (mgP gOM^{-1} h^{-1})	***K_S*** (mg P L^{-1})	***SPUA$_E$*** (L gOM^{-1} h^{-1})	
21-Oct-19	Medard	S	0.5	58.7 ± 7.5	271 ± 48	0.217	22.8 ± 8.9	53.6 ± 26.7	0.425 ± 0.072	0.51
21-Oct-19	Medard	S	1.5	5.7 ± 1.8	39.1 ± 16.3	0.146	–	–	0.078 ± 0.02	1.88
29-Apr-19	Most	N	0.5	47.7 ± 14.8	132 ± 48	0.361	40.8 ± 18.8	57.7 ± 34.4	0.708 ± 0.147	0.51
29-Apr-19	Most	N	1.5	50.2 ± 9.0	118 ± 24	0.427	58.7 ± 20.3	64.9 ± 27.8	0.905 ± 0.121	0.47
29-Apr-19	Most	S	0.5	72.1 ± 14.2	112 ± 31	0.643	129.2 ± 52.5	135.3 ± 62.9	0.955 ± 0.093	0.67
29-Apr-19	Most	S	1.5	59.5 ± 11.4	154 ± 40	0.386	62.1 ± 24.1	56.9 ± 27.8	1.092 ± 0.177	0.35
29-Jul-19	Most	N	0.5	5.7 ± 0.9	9.1 ± 2.4	0.628	6.5 ± 2.2	11.7 ± 6.4	0.554 ± 0.171	1.13
29-Jul-19	Most	N	1.5	7.0 ± 1.0	6.3 ± 1.5	1.112	6.3 ± 1.1	3.9 ± 1.5	1.594 ± 0.431	0.70
29-Jul-19	Most	S	0.5	5.1 ± 0.9	6.0 ± 2.1	0.841	4.7 ± 1	4 ± 1.8	1.155 ± 0.341	0.73
29-Jul-19	Most	S	1.5	7.8 ± 1.5	12.2 ± 4.4	0.638	6.5 ± 1.2	4.2 ± 1.7	1.54 ± 0.435	0.41
30-Sep-19	Most	N	0.5	6.1 ± 0.7	27.1 ± 4.4	0.224	4.4 ± 1.2	12.4 ± 5.3	0.359 ± 0.085	0.62
30-Sep-19	Most	N	1.5	13.7 ± 2.0	97.9 ± 16.6	0.14	7.7 ± 2	16.5 ± 6.5	0.468 ± 0.095	0.30
30-Sep-19	Most	S	0.5	6.6 ± 2.3	32.9 ± 14.8	0.202	3.4 ± 1	7.6 ± 3.9	0.448 ± 0.133	0.45
30-Sep-19	Most	S	1.5	5.1 ± 1.7	18.5 ± 9.1	0.277	–	–	0.169 ± 0.038	1.64
**Average**				**28.9**	**89.7**	**0.49**	**19.1**	**18.6**	**0.803**	
